# Effect of Very-Low-Calorie Ketogenic Diet on Psoriasis Patients: A Nuclear Magnetic Resonance-Based Metabolomic Study

**DOI:** 10.1021/acs.jproteome.0c00646

**Published:** 2020-11-09

**Authors:** Giuseppe Castaldo, Imma Pagano, Manuela Grimaldi, Carmen Marino, Paola Molettieri, Angelo Santoro, Ilaria Stillitano, Rocco Romano, Paola Montoro, Anna Maria D’Ursi, Luca Rastrelli

**Affiliations:** † NutriKeto_LAB Unisa−“San Giuseppe Moscati” National Hospital (AORN), Contrada Amoretta, 83100 Avellino, Avellino, Italy; ‡ Department of Pharmacy, University of Salerno, Via Giovanni Paolo II 132, 84084 Fisciano, Salerno, Italy

**Keywords:** psoriasis, obesity, ^1^H NMR metabolomics, very-low-calorie ketogenic diet, biomarkers

## Abstract

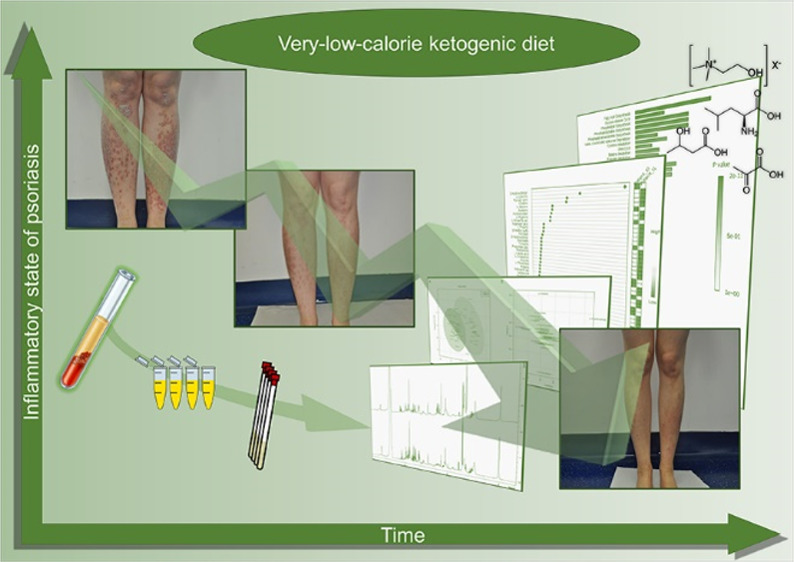

Psoriasis is an inflammatory disease of the epidermis based on an immunological mechanism involving Langerhans cells and T lymphocytes that produce pro-inflammatory cytokines. Genetic factors, environmental factors, and improper nutrition are considered triggers of the disease. Numerous studies have reported that in a high number of patients, psoriasis is associated with obesity. Excess adipose tissue, typical of obesity, causes a systemic inflammatory status coming from the inflammatory active adipose tissue; therefore, weight reduction is a strategy to fight this pro-inflammatory state. This study aimed to evaluate how a nutritional regimen based on a ketogenic diet influenced the clinical parameters, metabolic profile, and inflammatory state of psoriasis patients. To this end, 30 psoriasis patients were subjected to a ketogenic nutritional regimen and monitored for 4 weeks by evaluating the clinical data, biochemical and clinical parameters, NMR metabolomic profile, and IL-2, IL-1β, TNF-α, IFN-γ, and IL-4 concentrations before and after the nutritional regimen. Our data show that a low-calorie ketogenic diet can be considered a successful strategy and therapeutic option to gain an improvement in psoriasis-related dysmetabolism, with significant correction of the full metabolic and inflammatory status.

## Introduction

Psoriasis is a chronic inflammatory and multifaceted disease. This condition affects approximately 2–3% of the world’s population^1^ and is less common in children than in adults. Psoriasis is associated with morbidity and mortality.^2^ Generally, significant differences among individuals from various ethnic groups and geographical location have been recorded, with an increased incidence in individuals living at high latitudes.^3^ From a sex viewpoint, some studies reported differences between males and females.^4,5^ Patients with psoriasis have a decreased quality of life,^6^ with anxiety and depression. Psoriasis is a disorder of multifactorial etiology with both genetic and trigging factors. Numerous studies have reported the identification of genetic loci, in particular, 10 loci as susceptibility regions. The factors identified as triggers for the development of psoriasis are trauma, obesity, infections, medications, sunlight, stress, alcohol, smoking, and endocrine factors.^7^

Histologically, this disease is characterized by hyperproliferative keratinocytes and the infiltration of prominent T cells, dendritic cells, and neutrophils in the dermis. The diagnosis is usually clinical, including an examination of the primary lesion and affected areas. General presentations include plaque, inverse, guttate, erythrodermic, and pustular forms, with cutaneous manifestations and nail, scalp, and joint abnormalities. The psoriasis area and severity index (PASI), body surface area (BSA), and dermatology life quality index (DLQI) are tools that are commonly used for the classification of plaque psoriasis.^8^

A systematic review and meta-analysis summarize the epidemiological association between psoriasis and obesity, indicating a higher prevalence and incidence of obesity in psoriasis patients, compared with the general population.^9−11^ Moreover, numerous studies have reported evidence of a causal relationship between obesity and psoriasis, investigating the connection between body mass index (BMI) and psoriasis.^10,12,13^

A few studies of weight-loss interventions have been shown to improve psoriasis and to increase the response to treatment,^12^ especially adherence to a low-calorie dietary regimen.^14−17^ Fatty tissue is an active endocrine tissue and causes a pro-inflammatory state in obese patients.^18^ The clinical effect of weight loss is the reduction of adipose tissue as a source of pro-inflammatory cytokines. From this perspective, a very-low-calorie diet with adequate protein content is responsible for weight loss and the reduction of visceral fat mass.^19−22^

The ketogenic diet is a nutritional regimen characterized by a reduction in carbohydrates and a relative increase in protein and fat.^23^ At a biochemical level, the ketogenic diet induces a switch to ketone metabolism, causing a reduction in blood glucose and an increase in blood ketones and mitochondrial function.^24^ Recent scientific studies have shown the therapeutic potential of ketogenic diets in many diseases, such as diabetes, polycystic ovary syndrome, acne, neurological diseases, cancer, and the amelioration of respiratory and cardiovascular disease risk factors.^25−30^ Moreover, the ketogenic diet has been identified as an effective remedy for obesity and psoriasis, with a significant reduction in inflammatory components that are possibly localized in visceral adipose tissue.^17,22^

In recent years, metabolomic studies have played a significant role in revealing biomarkers, identifying the biochemical pathways involved in many diseases, and in providing information related to pathway perturbations. Nuclear magnetic resonance (NMR) spectroscopy represents a robust and suitable technique for metabolomic approaches: low-molecular-mass compounds can be concurrently qualitatively and quantitatively detected in biological samples.^31^ In this novel study, we performed an NMR-based metabolomic analysis of the sera of psoriasis patients subjected to a ketogenic nutritional regimen for 4 weeks. Metabolomic data analyzed with the aid of univariate and multivariate statistical methods were correlated with the biochemical and clinical parameters, including IL-2 and IL-1β cytokines. Metabolomic profiles of psoriasis patients compared to those of healthy controls before and after a 4 week ketogenic diet provide preliminary indications to identify candidate biomarkers useful in the theranostic control of psoriasis. Results of the metabolic pathway analysis reveal the therapeutic potential of a dietary regimen and provide new insights into the etiopathogenesis of psoriasis.

## Materials and Methods

### Participants

The study was conducted at the NutriKeto_LAB, Azienda Ospedaliera “San Giuseppe Moscati”, Avellino, Italy, between October 1, 2018, and March 1, 2019. Consecutive participants were recruited from hospital wards. Demographics and clinical information are reported in Table 1.

**Table 1:** This Table Describes the Demographics and Clinical Information of the Participants^a^

parameter	psoriasis group (*N* = 30)	control group (*N* = 30)	*P*
sex (male/female)	11/19	10/20	
age (mean ± SD, years)	42.8 ± 14.04	50.0 ± 9.90	0.003
BMI (kg/m^2^)	30.82 ± 5.96	28.4 ± 1.61	0.044
disease duration (mean ± SD, years)	5.09 ± 1.80	NA	
BSA (mean ± SD)	16.02 ± 3.39	NA	0.069^b^
PASI (mean ± SD)	8.69 ± 1.80	NA	0.007^b^
PsO/PsO + PsA	25/5	NA	
smokers	60%	50%	

a *P* value obtained by the Mann−Whitney *U* test between the psoriasis group and control group. BMI, body mass index; NA, not applicable; BSA, body surface area; PASI, psoriasis area and severity index; PsO, psoriasis; PsA, psoriatic arthritis.

b *P* value obtained by the Mann−Whitney *U* test of psoriasis group between T0 and T1.

### Inclusion and Exclusion Criteria

Participants eligible for inclusion criteria in the psoriasis group were overweight patients, aged 18–65 years old, with plaque psoriasis: 35 patients were screened, and 30 patients were recruited and completed the intervention study. The exclusion criteria were pregnancy; breastfeeding; insulin treatment; heart, kidney, or liver disease; obesity due to hypothyroidism; neoplastic disease; intentional or unintentional weight loss of more than 5 kg up to 3 months before the study; active treatment or treatment in the past 2 weeks with topical drugs or retinoic acid, ultraviolet light therapy or systemic therapy (in the recent 4 weeks), or biological preparations in the past 12 weeks; and recent history of drug addiction or alcohol abuse.

### Study Design

Blood serum samples were collected from 30 healthy subjects used as controls and 30 subjects diagnosed with psoriasis according to the mentioned psoriasis diagnostic criteria. The institutional ethical committee of Azienda Ospedaliera “San Giuseppe Moscati”, Avellino, Italy, approved the study protocol, which followed the 1964 Declaration of Helsinki and its later amendments, and all subjects gave written informed consent.

### Clinical Assessments

The diagnosis of plaque psoriasis was made by dermatologists based on clinical characteristics. PASI, BSA, the DLQI, and the VAS for itch ratings were employed to measure psoriasis severity. All the participants had height and body weight measured by calibrated flat scales equipped with a telescopic vertical steel stadiometer (SECA 711, Hamburg, Germany). BMI was calculated as the weight (kg) divided by the height squared (m^2^) kg/m^2^. A flexible plastic tape was used to assess waist and hip circumferences. Blood samples were analyzed in the clinical laboratory using automated analyzers and available commercial kits. Quantitative evaluation of the following clinical parameters was performed: hemoglobin, total lymphocyte count, creatinine, uric acid, glucose, insulin, C-peptide, glycated hemoglobin, growth hormone (GH), total cholesterol, high-density lipoprotein (HDL) cholesterol, low-density lipoprotein (LDL) cholesterol, triglycerides, apolipoproteins A1 and B (Apo A1 and Apo B, respectively), albumin, cholinesterase, serum aspartate aminotransferase (AST), alanine aminotransferase, gamma glutamyl transferase (γGT), lactate dehydrogenase, sodium, potassium, magnesium, calcium, phosphorus, the homeostasis model assessment insulin resistance (HOMA-IR), bilirubin, hematocrit, prothrombin activity, cortisol, vitamin B12, folic acid, azotemia, insulin, and homocysteine. For the assessment of visceral adipose tissue (VAT), ultrasound measurement of the aortomesenteric fat thickness (AMFT) was performed according to a previous procedure.^32^

### Dietary Intervention and Assessment

The recommendations for daily nutrient intake were met during the entire study time. The participants met a study dietician every week to verify food intake and adherence to administered dietary intervention. During group meetings, the diet regimen was given to subjects with encouragement and instructions for the use of dietary supplements. Treatment efficacy was assessed at baseline (T0) and after 4 weeks (T1). The control group (healthy controls) adopted a conventional diet and was instructed to eat ordinary food according to the national guidelines for a healthy diet.

Diet recommendations for psoriasis patients included the consumption of a very-low-calorie (<500 kcal/day) protein-based diet providing 10–20 g of carbohydrates (from vegetables, 400–500 g/day), 20–30 g of lipids, and 1.4 g per kg of ideal body weight (calculated using Lorentz’s equation) of protein per day. Half of this protein dosage is sufficient to supply 12 g of 90% whey protein, with the addition of l-arginine, α-ketoglutarate, l-ornithine, l-carnitine, l-glutamine, taurine, l-citrulline, l-cysteine, and vitamin B6. Other dietary supplements were alkalizing substances (calcium carbonate, magnesium citrate, potassium bicarbonate, potassium citrate, and sodium bicarbonate) and herbal remedies (with diuretic, anti-inflammatory, hepatoprotective, and antioxidant activities), such as garcinia (*Garcinia cambogia*), hawthorn (*Crataegus oxyacantha*), java tea (*Orthosiphon stamineus*), dandelion (*Taraxacum officinale*), thistle fruit extract (*Silybum marianum*), a multivitamin (C, D, K, and A)/multimineral supplement, and 10 g of hydrolyzed collagen powder. Ashwagandha (*Withania somnifera*) and bacopa (*Bacopa monnieri*) were also administered for psychophysical balance, and Triphala (*Phyllanthus emblica*, *Terminalia chebula*, and *Terminalia bellirica*) was administered to implement the correct intestinal function. Patients were advised to drink at least 2 L of bicarbonate-rich alkaline water per day (not tea or coffee) and to not use table salt but to salt their food with potassium chloride. All treatments with hypoglycemic agents and diuretics were interrupted.

### Outcomes (Primary and Secondary)

The primary outcome was PASI, an index of psoriasis severity: the PASI was measured at baseline (T0) and at 4 weeks (T1), along with BSA assessment. The secondary outcome was DLQI to determine the quality of life, the reduction in BSA, the improvement in itch severity, and weight loss.

### Sample Pretreatment for NMR Analysis

NMR sample preparation and NMR spectra acquisition were performed as previously reported.^31,33^ To obtain the blood serum, the whole blood was collected into tubes not containing anticoagulant and allowed to clot at room temperature for 30 to 120 min. After centrifugation at 12,000 *g*, the blood serum was aliquoted and stored at −80 °C in Greiner cryogenic vials before NMR spectroscopy measurements. Before being transferred to a 5 mm heavy-walled NMR tube, samples were thawed at room temperature. NMR samples were prepared by mixing 300 μL of blood sera with 200 μL of phosphate buffer, including 0.075 M Na_2_HPO_4_·7H_2_O, 4% NaN_3_, and H_2_O. Trimethylsilyl propionic-2,2,3,3-*d*_4_ acid, sodium salt (0.1% TSP in D_2_O) was used as an internal reference for the alignment and quantification of NMR signals; the mixture, homogenized by vortexing for 30 s, was transferred to a 5 mm NMR tube (Bruker NMR tubes) before analysis started.^31^

### NMR Data Acquisition

NMR experiments were carried out on a Bruker DRX600 MHz spectrometer (Bruker, Karlsruhe, Germany) equipped with a 5 mm triple-resonance *z*-gradient CryoProbe. TOPSPIN, version 3.0, was used for spectrometer control and data processing (Bruker Biospin, Fällanden, Switzerland). For nonfiltered biofluids, low-mass metabolites coexist with high-mass biomolecules, such as lipids, proteins, and lipoproteins; therefore, to selectively observe small-molecule components in solutions, Carr–Purcell–Meiboom–Gill (CPMG) experiments were performed. 1D ^1^H pulse-sequence CPMG experiments comprised a spectral width of 7 kHz, 32,000 data points, water presaturation applied over 3.5 s of relaxation delay, and a spin-echo delay of 80 ms.^34^ The pulse sequence used included an excitation sculpting routine for the suppression of the water signal.^35^ Due to the effect of excitation sculpting on the signal height of resonances in the region close to the water resonance,^36,37^ the metabolites that have resonances close to this region (ascorbate, glucose, mannose, and pyroglutamate) were quantified using resonances from those metabolites in other spectral regions. A weighted Fourier transform was applied to the time domain data with a 0.5 Hz line-broadening followed by a manual phase and baseline correction in preparation for targeted profiling analysis.

### NMR Data Processing

NMR spectra were manually phased and baseline-corrected. The quantification of serum metabolites was achieved using Chenomx NMR-Suite v8.0 (Chenomx Inc., Edmonton, Canada).^33,38^ Briefly, the Chenomx profiler is linked to the Human Metabolome Database (HMDB), containing more than 250 metabolite NMR spectral signatures encoded at different ^1^H spectrometer frequencies, including 600 MHz (http://www.hmdb.ca). A comparison of the spectral data obtained for each serum sample with the Chenomx metabolite library results in a list of compounds together with their respective concentrations based on the known concentration of the added internal reference compound TSP-*d*_4_ (5.8 mM).

### Statistical Analysis

Multivariate statistical analysis, principal component analysis (PCA), and partial least-squares discriminant analysis (PLS-DA) were conducted with normalized metabolomics data using MetaboAnalyst 4.0 (http://www.metaboanalyst.ca/). The performance of the PCA and PLS-DA model was evaluated using the coefficient *Q*^2^ (using the 7-fold internal cross-validation method) and coefficient *R*,^2^ defining the variance predicted and explained by the model, respectively. The loading plot was used to identify significant metabolites responsible for maximum separation in the PLS-DA score plot, and these metabolites were ranked according to their variable influence on projection (VIP) scores. VIP scores are weighted sums of squares of the PLS-DA weights, which indicate the importance of the variable.

### Quantitative Analysis

The data relative to the metabolite concentrations were analyzed using the PRincipal COmponent Normalization Algorithm (PRICONA),^39^ and the normalization strategy was applied.^40−43^ Accordingly, the proportional variations of ^1^H NMR signals were described by normalization factors, and the normalization constant was calculated as the score relative to the normalization setting.

### Cytokine Analysis: ELISA

Serum concentrations of the cytokines IL-2, IL-1β, TNF-α, IFN-γ, and IL-4 were determined by enzyme-linked immunosorbent assay (ELISA) commercial kits (Diaclone SAS (Besançon Cedex, France)) according to the manufacturer’s instructions. All tests were performed in duplicate. The ranges of the sensitivity standard curve of the ELISA kits were 31.2–1000 pg/mL for IL-2, 15.6–500 pg/mL for IL-1β, 25–800 pg/mL for TNF-α, 12.5–400 pg/mL for IFN-γ, and 1.1–35 pg/mL for IL-4.

Standard diluents, the capture antibody, and the detection antibody were obtained as a complete kit for each cytokine. Standard diluent–serum samples were used to obtain a Cedex standard curve. The samples in the multiwell plate were mixed by repeated aspirations and ejections, taking care not to scratch the inner surfaces. Freeze-dried control vials were also reconstituted with the most appropriate standard diluent to the sample to gain a solution at the concentration stated on the vial. Biotinylated anticytokines, biotinylated secondary antibody, and streptavidin-HRP were also prepared according to the manufacturer’s protocol. The absorbance value of each well was read on a Thermo Scientific Multiskan GO spectrophotometer using 450 nm as the primary wavelength and 620 nm as the reference wavelength (610 to 650 nm is acceptable).

## Results

### Clinical Analysis

Clinical data for 30 patients with psoriasis before and after the ketogenic diet were statistically analyzed using the Mann–Whitney *U* test to assess their significance and *P* value. The VIP score was calculated using the R package to identify the variables that discriminated between the two groups. The variables with VIP scores >1 were considered significant for the analysis. The VIP score values for selected variables concerning psoriasis patients before and after the ketogenic diet are given in Table 2.^44^

**Table 2:** VIP Score and *P* Value Relative to Clinical Features Calculated by the R Package

parameter	VIP score	T0	T1	*P*^a^
DLQI	2.2108	+	–	1.3 × 10^–05^
folic acid	1.9214	–	+	4.3 × 10^–05^
VAS pain	1.7323	+	–	3.3 × 10^–03^
VAS pruritus	1.7504	+	–	2.5 × 10^–03^
vitamin B12	1.7297	–	+	6.3 × 10^–04^
AST	1.7140	–	+	3.4 × 10^–02^
LDL	1.6913	+	–	8.7 × 10^–06^
cortisol	1.6028	–	+	1.3 × 10^–02^
PASI	1.4916	+	–	7.0 × 10^–03^
calcium	1.3501	–	+	5.3 × 10^–03^
total cholesterol	1.2921	+	–	4.3 × 10^–06^
direct bilirubin	1.2533	–	+	1.9 × 10^–02^
HOMA-IR	1.2406	+	–	2.8 × 10^–03^

a *P* value calculated by the Mann–Whitney *U* test.

Table 2 shows clinical and biochemical clinical parameters for psoriasis patients. An analysis of the data indicated that all parameters related to psoriasis improved after the ketogenic diet. In particular, the DLQI, visual analogue scale (VAS) pruritus, VAS pain, and PASI improved, indicating that the ketogenic diet reduced psoriasis symptoms.^45,46^ Clinical data identified by the VIP score also included several biochemical and clinical parameters derived from blood analysis. In particular, the concentrations of folic acid, vitamin B12, AST, cortisol, calcium, and direct bilirubin were higher in patients at T1 than at T0. LDL cholesterol, total cholesterol, and HOMA-IR levels were lower in patients at T1 than at T0 (see Table 2).

### Quantitative Analysis

**Figure 1:**
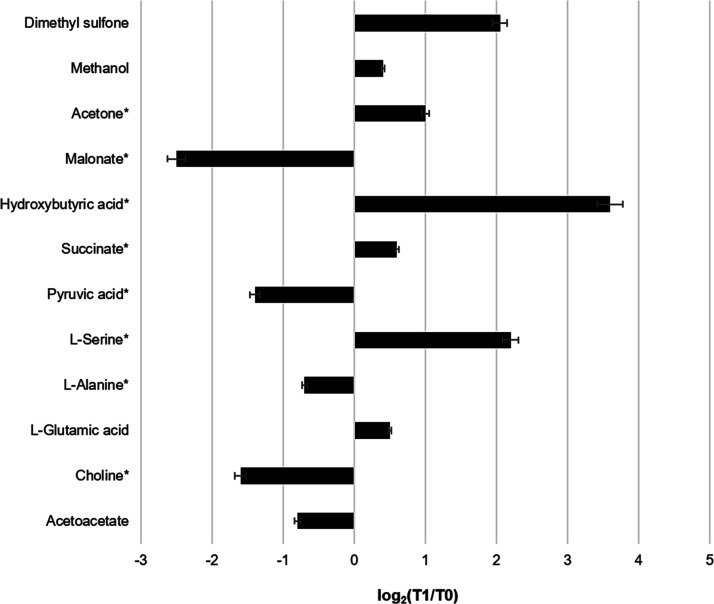
Histogram summarizing the fold change as log_2_(T1/T0) in the various metabolites quantified after the normalization procedure. Fold changes are obtained by comparing the means of the metabolite signals of 30 psoriasis patients at T0 to those at T1. Positive differences are relative to overexpressed metabolites at T1 with respect to T0. The differences were considered significant (*P* < 0.05). Metabolites with a significant difference are marked with asterisks.

To quantitatively evaluate the ^1^H NMR spectra of the serum of participants at T0 in comparison to the NMR spectra of the serum of the same participants at T1, the PRincipal COmponent Normalization Algorithm (PRICONA) and normalization strategy were applied. The PRICONA and normalization strategy are based on the assumption that, since concentration differences result in proportional variations of spectral intensities, nonproportional changes most likely can be attributed to the effects of the disease. The proportional variations are described by the normalization factor *R*, which must be calculated to compare the intensities of the T0 and T1 signals. When the first principal component (PC) explains the major part of the variance of a spectral data set, it represents the function shape of the sum of a group of proportional peaks (normalization set), and its scores represent the proportionality constants (normalization constants). Once the spectra are normalized, peak intensities can be directly compared. According to the normalization procedure described above, signals that showed significant variations were quantified. At the same time, an opportune strategy based on the PRICONA is performed to achieve a reliable NMR metabolomic analysis. The normalization algorithm is based on PCA with some advantages: it allows simultaneous normalization of data sets of spectra by identifying signals affected by the agent (in this case, after the diet regimen) and quantitative measurement of variations. In this way, the differences in peak intensities are excluded, and the real differences do not depend on differences in sample concentration. All extraneous sample-to-sample variations and those within each metabolite are removed. After spectral normalization, all differences related to metabolites can be used to identify potential candidate biomarkers. Figure 1 shows the most significant differences observed in the signals of the spectra at T1 compared to those at T0: the variations in concentration are indicated as log_2_ (fold change); the differences characterized by *P* < 0.05 are considered significant. An analysis of the data indicated a significant increase in l-serine, dimethyl sulfone, and hydroxybutyric acid and a decrease in malonate, choline, and pyruvic acid.

### Multivariate Data Analysis

**Figure 2:**
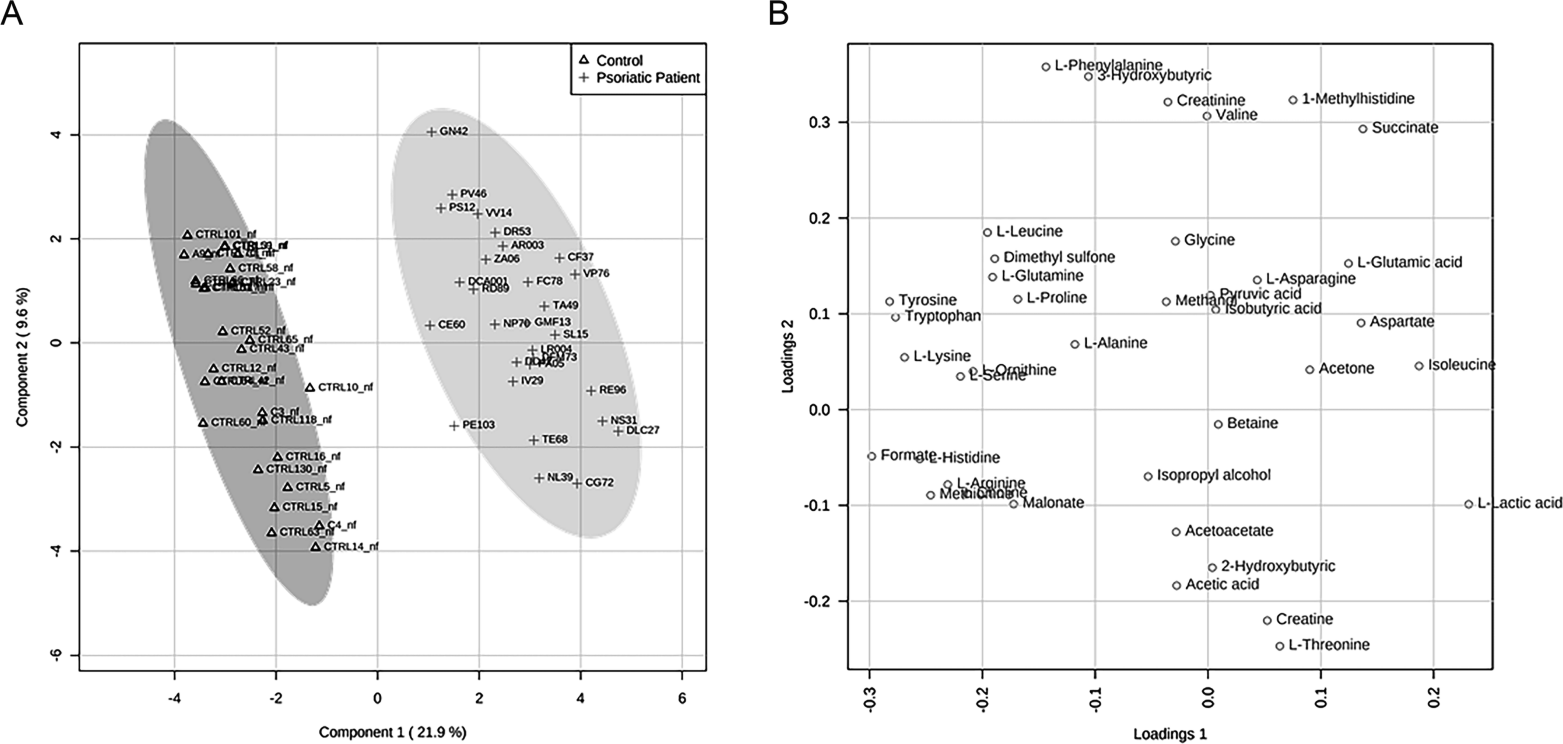
PLS-DA score scatter plot (A) and PLS-DA loading scatter plot (B) for the ^1^H NMR data collected in 1D-^1^H-CPMG spectra acquired at 600 MHz. Data represent the sera from 30 controls and 30 psoriasis patients before the ketogenic diet.

Matrices, including metabolites and their concentrations as derived from ^1^H NMR data collected in 1D-^1^H-CPMG,^34^ were analyzed according to multivariate statistical analysis using MetaboAnalyst 4.0.^47^ Multivariate analysis (MVA) was performed to identify the metabolic profile of psoriasis patients. The original matrix included the sera from 30 subjects with psoriasis and the sera from 30 healthy controls. The data matrix, after normalization by sum, log transformation, and Pareto scaling, was analyzed by PLS-DA (Figure 2). To minimize false discoveries and to obtain robust statistical models, *t*-tests and fold-change tests were applied according to good standardized practice (see Tables S1 and S2, Supporting Information).^47^ In Figure 2, PLS-DA shows that the data sets relative to psoriasis sera are well separated from control sera. The first component explains 21.9% of the variance, while the second explains 9.6%.

**Figure 3:**
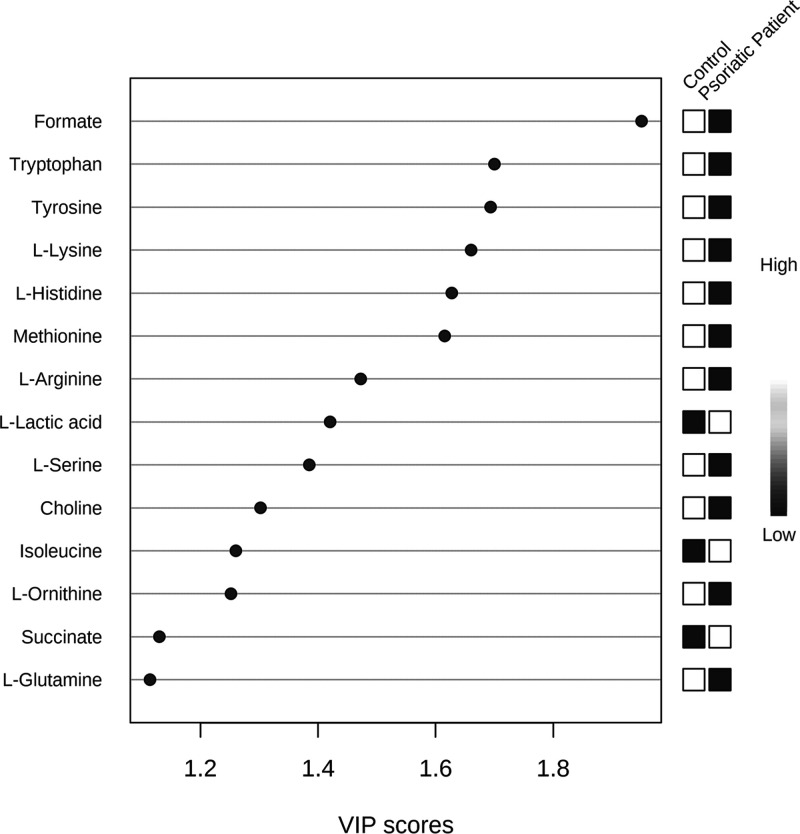
Metabolites discriminating healthy controls from psoriasis patients before the ketogenic diet according to VIP score values.

The creation of separate clusters indicates a different metabolome characterizing patients with psoriasis and healthy controls. This evidence is confirmed by applying VIP score analysis (Figure 3). Accordingly, the metabolites characterized by a VIP score higher than 1 are considered good classifiers between psoriasis patients and healthy controls. The metabolites considered as discriminative of the metabolomes of controls and those of subjects with psoriasis are represented in the VIP score graph in Figure 3. In particular, we observed an amino acid dysmetabolism correlated to a lower concentration of l-tryptophan, l-tyrosine, l-lysine, l-histidine, l-methionine, l-arginine, l-ornithine, and l-glutamine in psoriasis patients.

The multivariate analysis was repeated, taking into account patients before ketogenic diet (T0) and after the 4 week ketogenic diet (T1). Original matrices were normalized according to the concentration ranges of HMDB.^48^ The data matrix, after normalization by sample median and log transformation, was analyzed by PCA (see Figures S1 and S2, Supporting Information) and PLS-DA (see Figure S3, Supporting Information). To obtain robust statistical models and to calculate the *P* value, the Mann–Whitney *U* test was applied.^49^ For each sample, 38 metabolites were identified and quantified.

**Figure 4:**
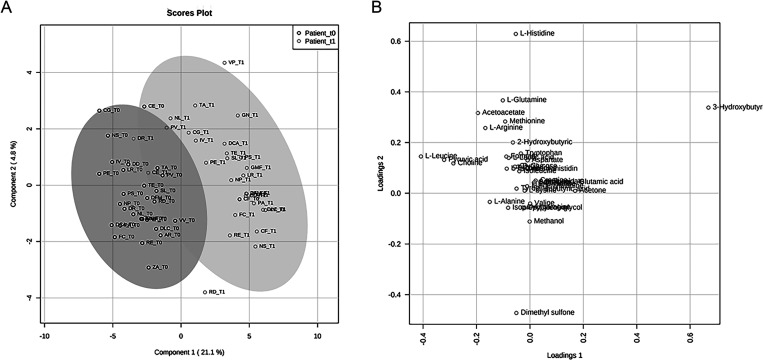
PLS-DA score scatter plot (A) and PLS-DA loading scatter plot (B) for the ^1^H NMR data collected in the 1D-^1^H-CPMG spectra acquired at 600 MHz. Data are relative to sera of 30 psoriasis patients at T0 (before ketogenic diet) and those of 30 psoriasis patients at T1 (after 4 weeks of the diet).

**Figure 5:**
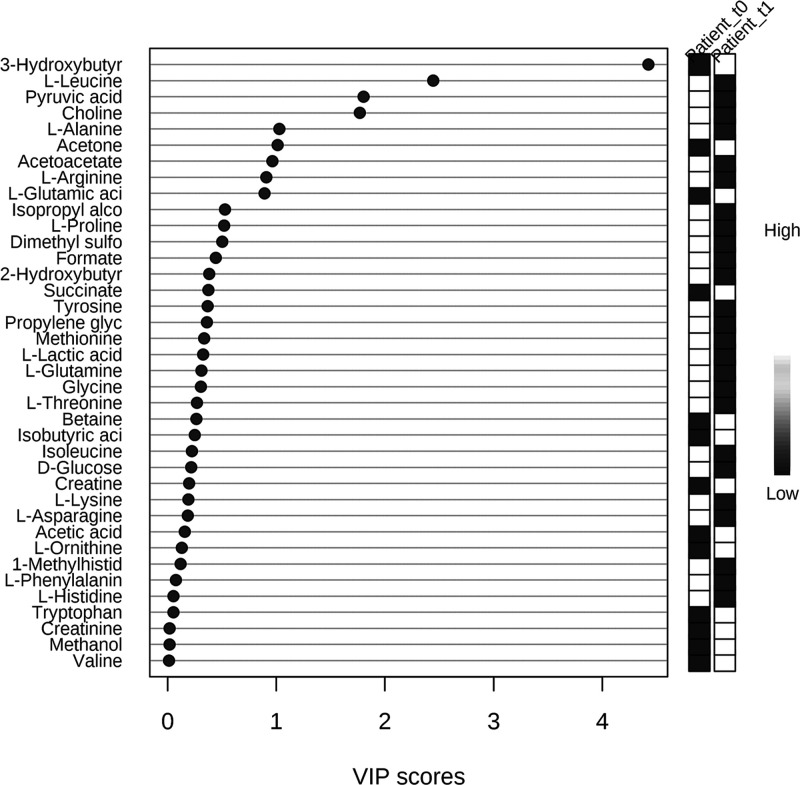
Metabolites discriminating psoriasis patients at baseline (T0) from psoriasis patients after 4 weeks of the diet (T1) according to VIP score values. Only metabolites with VIP score > 1 are discriminant between patients before diet (T0) and psoriasis patients after 4 weeks of the diet (T1).

An inspection of the PLS-DA score scatter plot (Figure 4A) and loading scatter plot (Figure 4B) points to 3-hydroxybutyrate, l-leucine, pyruvic acid, and choline as metabolites that significantly discriminate patients at baseline from those after 4 weeks of the diet. This evidence is confirmed through the application of VIP score analysis (Figure 5) (for details, see Table S3, Supporting Information). In particular, the graph reported in Figure 5 shows that, before the diet, psoriasis patient sera contain a higher concentration of l-leucine, pyruvic acid, choline, l-alanine, and acetoacetate and a lower concentration of 3-hydroxybutyrate and acetone than after the diet.

The results shown in Figure 5 are in agreement with those of the quantitative analysis regarding the significant metabolite identification, except for l-leucine. Furthermore, regarding the trends of the concentrations of significant metabolites before and after the diet, there are concordances with the PRICONA analysis, such as the increase in hydroxybutyric acid and the decrease in choline and pyruvic acid at T1 (see Figure 1).

To gain meaningful insight from these data, we applied metabolic pathway analysis using MetaboAnalyst 4.0.^47^ Similar to the MVA, we carried out pathway analysis on two clusters: (i) controls against psoriasis patients before the ketogenic diet and (ii) psoriasis patients before the ketogenic diet (T0) against psoriasis patients after 4 weeks from the ketogenic diet (T1). Table 3 shows that the results of the pathway analysis come from the comparison between patients at T0 (before the ketogenic diet) and controls and the comparison between psoriasis patients at T0 and psoriasis patients at T1 (after 4 weeks of diet). The *P* value and false discovery rate (FDR)^50^ are reported to confirm the significance of the pathways. We also reported the most discriminating metabolite (*P* < 0.05) belonging to the pathway and detected through the KEGG database.^51^

**Table 3:** Metabolic Pathway Analysis Related to the Comparison between Control and Psoriasis Patients at T0 and the Comparison between Psoriasis Patients at T0 and Psoriasis Patients at T1 (after 4 Weeks of Diet)^a^

pathway (control *vs* psoriasis patients before ketogenic diet)	raw *P*	FDR	discriminant metabolites	C	P
arginine and proline metabolism	2.59 × 10^–25^	9.58 × 10^–25^	arginine (*P =* 3.34 × 10^–08^)	+	–
			ornithine (*P* = 6.85 × 10^–06^)	+	–
			proline (*P* = 0.0020)	+	–
aminoacyl-tRNA biosynthesis	3.86 × 10^–19^	7.14 × 10^–18^	histidine (*P* = 2.14 × 10^–10^)	+	–
			lysine (*P* = 6.08 × 10^–11^)	+	–
			tryptophan (*P* = 1.18 × 10^–11^)	+	–
			tyrosine (*P* = 1.56 × 10^–11^)	+	–
glyoxylate and dicarboxylate metabolism	3.69 × 10^–11^	4.55 × 10^–10^	formate (*P* = 4.45 × 10^–18^)	+	–
			l-glutamine (*P* = 8.81 × 10^–05^)	+	–
			l-serine (*P* = 3.42 × 10^–07^)	+	–
histidine metabolism	3.79 × 10^–08^	3.51 × 10^–07^	histidine (*P* = 2.14 × 10^–10^)	+	–
arginine biosynthesis	9.39 × 10^–08^	6.38 × 10^–07^	arginine (*P* = 3.34 × 10^–08^)	+	–
cysteine and methionine metabolism	1.21 × 10^–07^	6.38 × 10^–07^	methionine (*P* = 3.36 × 10^–10^)	+	–
β-alanine metabolism	5.18 × 10^–07^	2.05 × 10^–06^	histidine (*P* = 2.14 × 10^–10^)	+	–
alanine, aspartate, and glutamate metabolism	5.94 × 10^–04^	1.83 × 10^–03^	l-glutamine (*P* = 8.81 × 10^–05^)	+	–
			succinate (*P* = 6.71 × 10^–05^)	–	+
glycine, serine, and threonine metabolism	1.14 × 10^–03^	3.24 × 10^–03^	choline (*P* = 2.36 × 10^–06^)	+	–
phenylalanine, tyrosine, and tryptophan biosynthesis	1.73 × 10^–03^	4.27 × 10^–03^	tyrosine (*P* = 1.56 × 10^–11^)	+	–

pathway (psoriasis patients before ketogenic diet *vs* psoriasis patients after ketogenic diet)	raw *P*	FDR	discriminant metabolites	P_T0_	P_T1_
synthesis and degradation of ketone bodies	2.27 × 10^–13^	4.33 × 10^–12^	3-hydroxybutanoate (*P* = 1.96 × 10^–13^)	**–**	**+**
butanoate metabolism	2.28 × 10^–13^	4.33 × 10^–12^	3-hydroxybutanoate (*P* = 1.96 × 10^–13^)	**–**	**+**
			l-glutamate (*P* = 0.008)	**–**	**+**
glycine, serine, and threonine metabolism	7.95 × 10^–03^	7.55 × 10^–02^	choline (*P* = 2.11 × 10^–07^)	**+**	**–**
			pyruvate (*P* = 4.21 × 10^–05^)	**+**	**–**
arginine and proline metabolism	4.19 × 10^–06^	3.11 × 10^–05^	proline (*P* = 0.007)	**+**	**–**
			pyruvate (*P* = 4.21 × 10^–05^)	**+**	**–**
alanine, aspartate, and glutamate metabolism	3.94 × 10^–06^	3.11 × 10^–05^	l-alanine (*P* = 0.0019)	**+**	**–**
			pyruvate (*P* = 4.21 × 10^–05^)	**+**	**–**
			l-glutamate (*P* = 0.008)	**–**	**+**
aminoacyl-tRNA biosynthesis	8.84 × 10^–06^	4.20 × 10^–05^	l-alanine (*P* = 0.0019)	**+**	**–**
			leucine (*P* = 1.96 × 10^–11^)	**+**	**–**
			l-glutamate (*P* = 0.008)	**–**	**+**
			proline (*P* = 0.007)	**+**	**–**
valine, leucine, and isoleucine degradation	7.95 × 10^–06^	4.20 × 10^–05^	leucine (*P* = 1.96 × 10^–11^)	**+**	**–**
valine, leucine, and isoleucine biosynthesis	1.16 × 10^–05^	4.87 × 10^–05^	leucine (*P* = 1.96 × 10^–11^)	**+**	**–**
pyruvate metabolism	1.59 × 10^–05^	4.87 × 10^–05^	pyruvate (*P* = 4.21 × 10^–05^)	**+**	**–**

a The *P* value, FDR value, and the most significant metabolites with the concentration variation related to the clusters taken in examination are reported for each pathway.

The comparison between controls and patient at T0 identified an amino acid dysmetabolism related to different pathways, in particular, arginine and proline metabolism; histidine metabolism; arginine biosynthesis; cysteine and methionine metabolism; alanine, aspartate, and glutamate metabolism; glycine, serine, and threonine metabolism; and phenylalanine, tyrosine, and tryptophan biosynthesis. In addition, the comparison between psoriasis patients at T0 and psoriasis patients at T1 highlighted the involvement of pathways related to ketogenic diet, in particular, synthesis and degradation of ketone bodies and butanoate metabolism, which are not disregulated in comparison with previous clusters. Also, in the comparison between psoriasis patients at T0 psoriatic patients at T1, there is an amino acid dysmetabolism related to glycine, serine, and threonine metabolism; arginine and proline metabolism; alanine, aspartate, and glutamate metabolism; and valine, leucine, and isoleucine degradation and biosynthesis.

**Figure 6:**
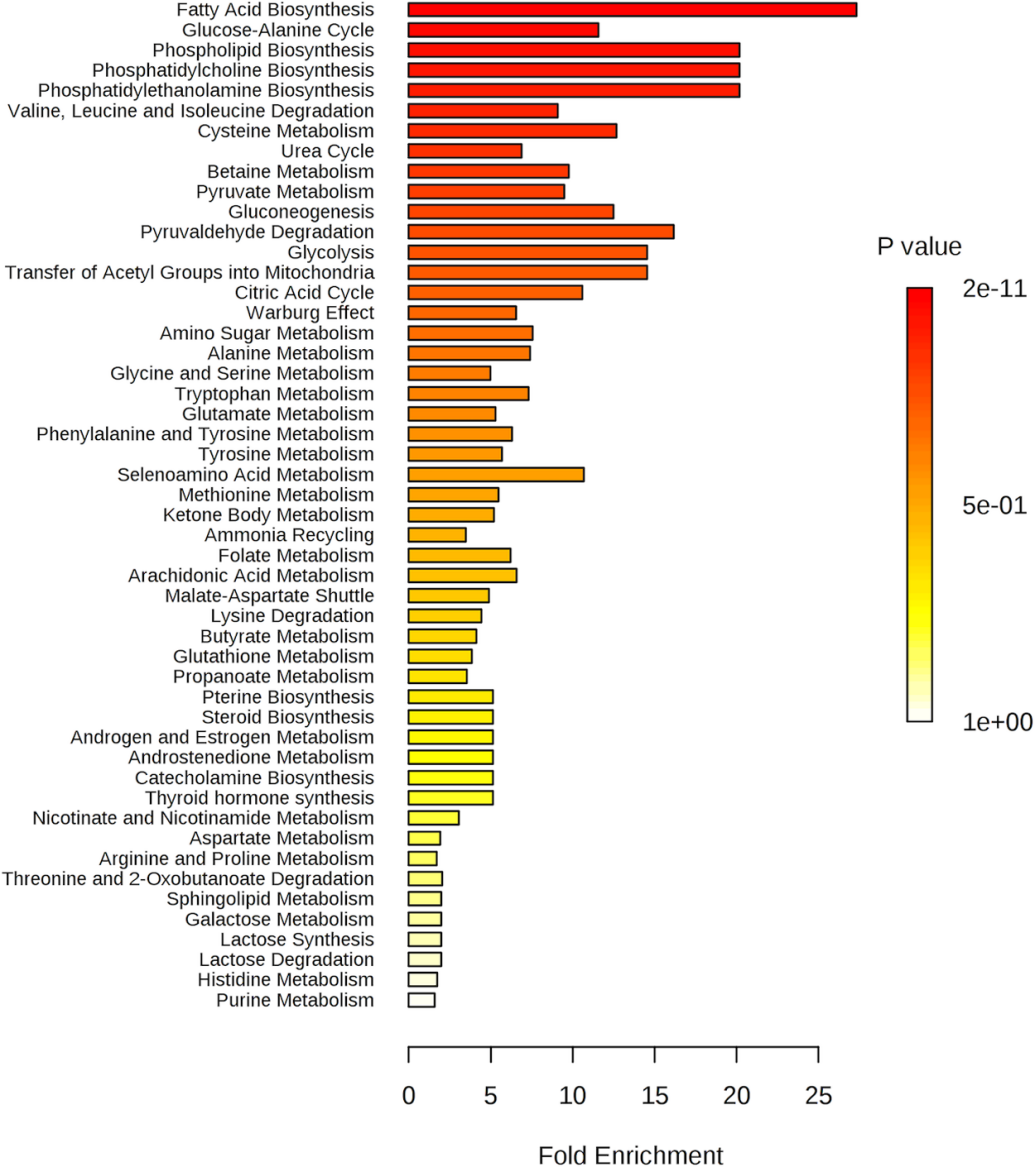
Pathway enrichment analysis: the pathways related to a *P* value that excludes randomness and is correlated with psoriasis.

In enrichment analysis, by increasing the number of reference metabolites and evaluating concentrations of metabolites present in our matrix, it is possible to identify a change in the metabolome of psoriasis patients before and after the diet regimen. All dysmetabolism identified in the pathway analysis was confirmed by the enrichment analysis (see Table S4, Supporting Information). The graph in Figure 6 shows the pathways that best discriminate between the two classes (T0 and T1). In particular, in the pathway of fatty acids, there is a physiological variation of the ketone bodies related to the ketogenic diet, with an increase of 3-hydroxybutyrate and acetic acid in the serum of patients after 4 weeks. Choline pathways (phospholipid biosynthesis and phosphatidylcholine biosynthesis) indicated a reduction in serum choline levels of patients with post-diet psoriasis.^52^ Amino acid dysmetabolism was confirmed by a change in l-leucine, l-isoleucine, and l-valine levels, which decreased after the diet,^53^ and by the dysfunction of the urea cycle.^53^

**Figure 7:**
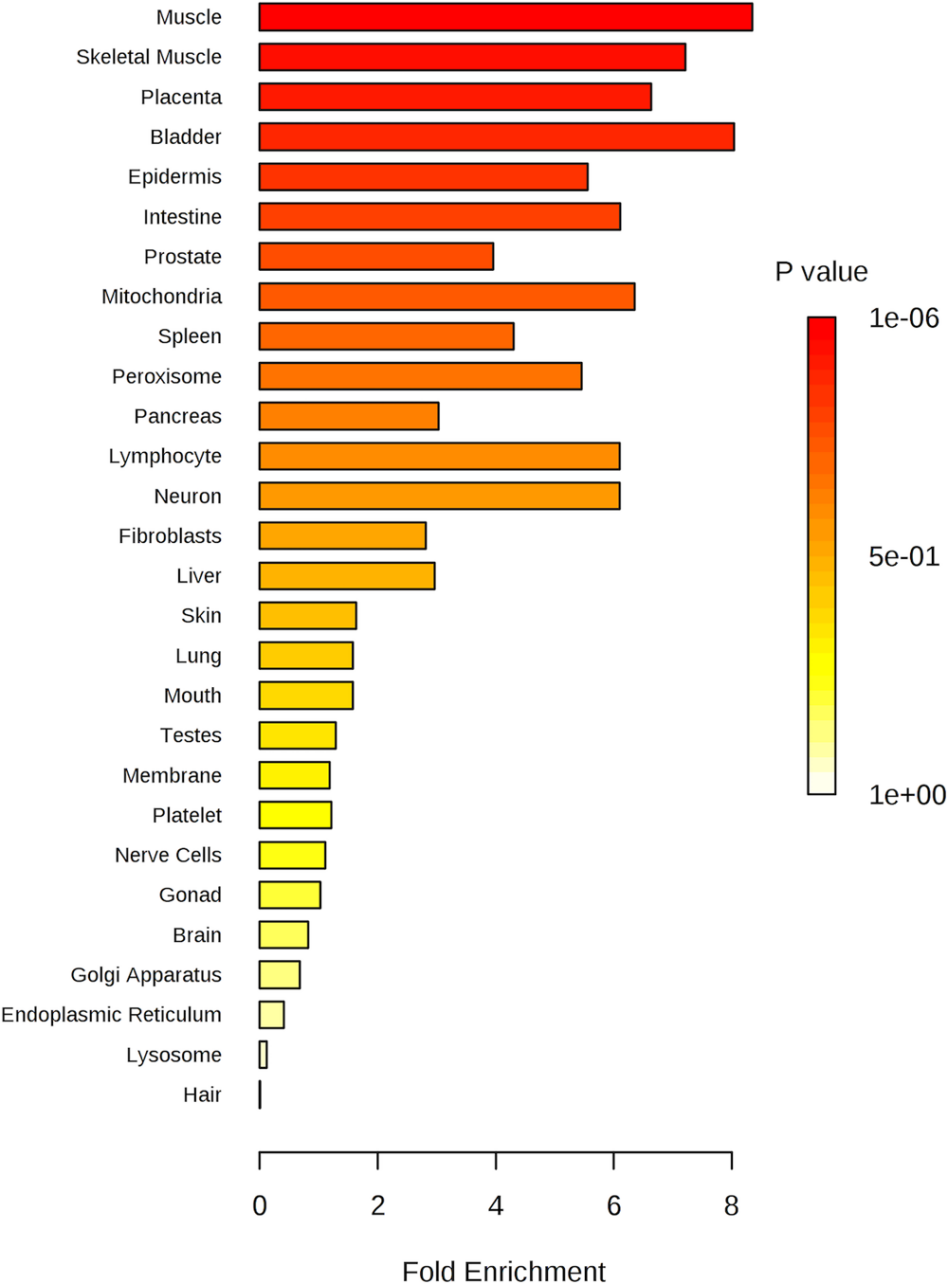
Metabolite set enrichment overview: the tissue related to a *P* value that excludes randomness and correlates with psoriasis.

Enrichment analysis was repeated using the parameter “location-based metabolite set” to understand which tissue was the most involved in the previously described dysmetabolism. The graph in Figure 7 represents the output of the enrichment analysis that showed a dysmetabolism related to the epidermis, muscular tissue, and skeletal muscle tissue, which is already known to be involved in the physiopathogenesis of psoriasis.^1^

A more accurate analysis of the metabolic pathways involved in psoriasis was carried out by Reactome analysis^54^ (see Table S5, Supporting Information). Pathway analysis by Reactome confirmed all dysmetabolism individuated to pathway analysis by MetaboAnalyst 4.0 and has shown a possible link to *SLC6A14* gene variations that may be associated with obesity. Several studies have confirmed the correlation between obesity and psoriasis.^10,12,13^

To assess the benefits of ketogenic diet for patients with psoriasis, the average of the concentrations of metabolites with VIP score > 1 was calculated in the following groups: (i) healthy controls, (ii) patients with psoriasis at T0 (before the ketogenic diet), and (iii) patients with psoriasis at T1 (after 4 weeks of diet). Table 4 shows the average metabolite values for each cluster and the difference between the metabolite’s mean concentration of healthy controls and patients before and after the diet. Analysis of the data shown in Table 4 indicates that the mean concentration difference of the metabolites in the patients *vs* controls at T1 is lower than the mean concentration difference in the patients *vs* controls at T0. The metabolites formate, l-histidine, methionine, l-arginine, choline, l-ornithine, pyruvic acid, and l-alanine are the most discriminant.

**Table 4:** Mean Discriminant Metabolites (VIP Score > 1) Concentration and Difference Mean Concentration Relative to Controls, Psoriasis Patients before Diet (T0), and Psoriasis Patients after 4 Weeks of Diet (T1)^a^

metabolites (VIP > 1)	M[]ctrl (μM)	M[]p(T0) μM	M[]p(T1) (μM)	M[]ctrl – M[]p(T0)	M[]ctrl – M[]p(T1)
formate	143.34	206.62	162.48	–65.76	–19.14
tryptophan	107.41	203.79	214.17	–96.38	–106.76
tyrosine	43.79	103.03	83.41	–59.24	–39.62
l-lysine	129.48	116.48	186.24	13	–56.76
l-histidine	303.27	348.10	300.10	–44.83	3.17
methionine	74.72	118.45	50.18	–43.71	24.56
l-arginine	17.83	53.31	14.00	–35.48	3.83
l-lactic acid	1235.86	13,490.45	10,661.03	–12,254.59	–9425.17
choline	39.83	116.86	39.41	–77.03	0.42
isoleucine	171.72	2507.27	1912.76	–2335.55	–1741.04
l-ornithine	70.17	136.96	91.41	–66.79	–21.24
succinate	12.69	401.03	509.34	–388.34	–496.65
l-glutamine	743.69	2087.62	2079.38	–1343.93	–1335.69
3-hydroxybutiric acid	69.34	385.90	4526.07	–316.56	–4456.73
l-leucine	21.48	101.48	19.62	–80	1.86
pyruvic acid	52.48	410.93	123.62	–358.45	–71.14
l-alanine	182.48	662.55	410.14	–480.07	–227.66
acetone	147.96	1336.14	2800.55	–1188.17	–2652.59

a M[]ctrl, mean concentration in control group; M[]p(T0), mean concentration in psoriasis patients at T0; M[]p(T0), mean concentration in psoriasis patients at T1.

### ELISA Results

**Figure 8:**
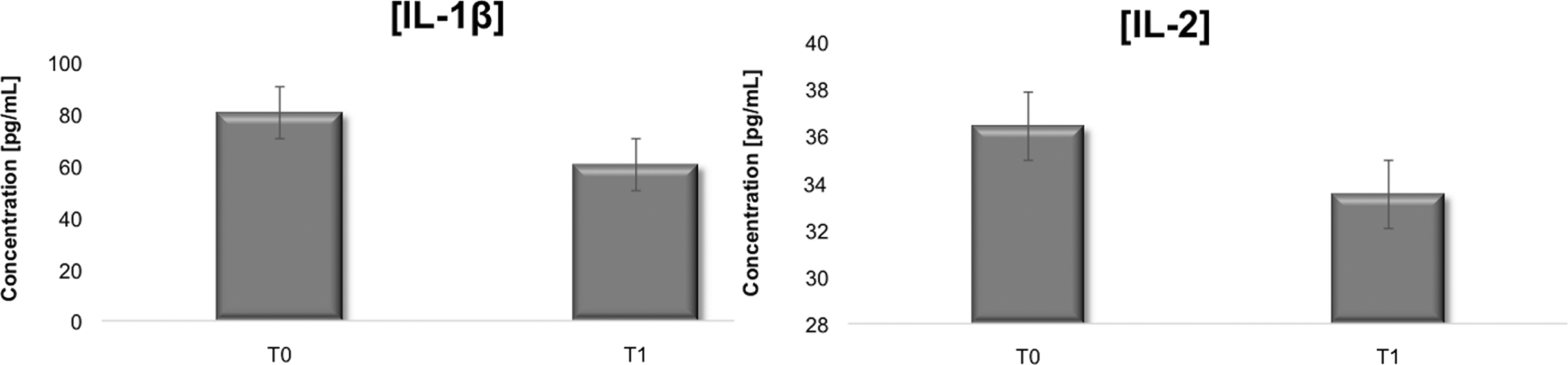
Mean resultant significant cytokine concentrations (*P* < 0.05) at T0 (before diet) and T1 (after 4 weeks of the diet).

ELISAs performed on serum samples to evaluate the levels of IL-2, IL-1β, TNF-α, IFN-γ, and IL-4 showed that there were significant differences in the levels of the cytokines IL-2 (*P* = 0.04) and IL-1β in patients between T0 and T1 (*P* = 0.006) (Figure 8). The significance of the data was assessed by the Wilcoxon statistical analysis test.^55^ The graph in Figure 8 represents the significant difference in mean cytokine concentrations for the patient group before the ketogenic diet (T0) and after 4 weeks of the diet (T1). No significant changes in concentrations were detected for TNF-α (*P* = 0.47), IFN-γ (*P* = 0.17), and IL-4 (*P* = 0.65).

## Discussion

In the present study, we evaluated the effect of a ketogenic diet on psoriasis disease progression. Thirty psoriasis patients were subjected to a ketogenic nutritional regimen and monitored by evaluating (i) the clinical symptoms, (ii) the blood biochemical parameters, including IL-2, IL-1β, TNF-α, IFN-γ, and IL-4, and (iii) the metabolomic profile, as derived from ^1^H NMR analysis. As a preliminary step, we identified the psoriatic patients’ metabolomic profile and the healthy controls’ metabolomic profile.^1^ Based on preliminary data previously obtained in our laboratory and for reasons related to the restrictions imposed from the nutritional regimen, the patients were subjected to 4 week treatment. As the parallel evaluation of ketogenic diet effects in psoriatic and healthy subjects was not possible, we considered the healthy controls’ metabolomic profile as our experimental control. By following the indications of the Italian Society of Endocrinology (ISE),^56^ the enrollment of healthy subjects for the low-calorie ketogenic nutritional regimen is not recommended as the low-calorie ketogenic diet causes alteration of the metabolic profile in healthy subjects, with potentially harmful implications in blood pH equilibrium, calcium homeostasis, and lean mass balance. Therefore, we considered the therapeutic effect of ketogenic diets by comparing the metabolomic profile of psoriatic patients at T1 with healthy subjects’ metabolomic profile at T0.

Data resulting from the clinical evaluation showed caloric restriction-induced psoriasis disease regression after 4 weeks with a significant reduction in the DLQI, PASI, VAS pain, and VAS pruritus clinical scores (*P* < 0.05). In particular, the PASI showed a reduction of approximately 50%.

Biochemical and clinical parameters indicated a general improvement in the metabolites’ concentration known to be related to the condition of psoriasis:^57^ folic acid, vitamin B12, calcium, bilirubin, cortisol, LDL, and total cholesterol. High levels of folic acid and vitamin B12 are known to improve the pathological condition related to psoriasis.^57^ As reported in Table 2, our data indicated low levels of folic acid and vitamin B12 at T0, whereas an increase in the concentration was registered at T1. Hypocalcemia was observed as a risk factor in psoriasis.^58^ Our data show an increase in calcium levels in subjects after the ketogenic diet. Bilirubin, an essential antioxidant metabolite, was present in low concentrations in subjects with psoriasis;^59^ after our diet regimen, bilirubin concentration increased. Significant variation was observed in the concentrations of cortisol, LDL, and total cholesterol. Recent scientific studies assert that low cortisol levels are related to high stress levels in subjects with psoriasis.^60^ According to the DLQI, investigating the quality of life in subjects with psoriasis, cortisol levels following a low-calorie ketogenic diet increased.

The ketogenic diet resulted in weight loss at T1, corresponding to ∼10% of the initial body weight. Other significant modifications of anthropometric measurements were waist circumference and hip circumference (see Table S6, Supporting Information). The weight loss was associated with a significant increase in ketone bodies at T1, as shown in the NMR-based metabolomic analysis. This effect was the main physiological effect of the ketogenic diet. The basic principle of the low-calorie ketogenic diet is to limit the availability of carbohydrates, forcing the consumption of fats as the primary energy source, with a resulting increase in fatty acids, ketone bodies, and pyruvic acid. However, weight loss and the increase in ketone bodies were not associated with any alteration of those biochemical parameters that initially were in the average concentration range, thus proving the safety of the proposed dietary intervention.

In contrast, biochemical and clinical parameters previously found in abnormal concentrations and related to carbohydrate and lipid metabolism, such as glucose, total cholesterol, LDL, Apo A1 and B, AST, γGT, insulin, and HOMA-I, returned to healthy average ranges (see Table S6, Supporting Information). A decrease in HDL was also observed due to the drop in total cholesterol (from 52.07 ± 18.23 to 44.20 ± 14.22 mg/dL; *P* = 0.079). Last, the reduction in the aortomesenteric fat thickness (AMFT) proved a substantial decrease in VAT.

The ketogenic nutritional regimen aims to minimize insulin levels and to increase GH secretion. The final effect is the almost complete reduction of the visceral adipose tissue responsible for insulin resistance and the insurgence of inflammatory status. Insulin and estrogen act at the PPAR (peroxisome proliferator-activated receptor) level by activating the transcriptase for adipogenesis. Conversely, GH phosphorylates PPAR and inhibits adipogenesis. As a result, reduced insulin concentration favors a lipolytic route with the mobilization of visceral localized fat deposits.^61−63^

Earlier scientific studies have identified lactic acid as a possible biomarker of psoriasis.^53,64^ Confirming this evidence, the VIP analysis (Figure 3) indicated increased lactic acid concentration in psoriatic patients. On the contrary, the concentrations of lactic acid and l-isoleucine, although decreasing, do not fall into the physiological range of healthy subjects, perhaps due to the short-term treatment.

The confirmation that the ketogenic diet induces a correction of the dysmetabolic condition related to psoriasis disease results from the NMR metabolomics study performed on psoriasis patients’ blood sera. NMR metabolomics data on the patient sera collected before the diet indicated abnormal concentrations of metabolites that are related to the condition of psoriasis,^65^ and these concentrations were found in the ranges of the healthy controls at T1, suggesting a rebalancing of the metabolome after the ketogenic regimen.^65^

Abnormally high concentrations of l-alanine and l-leucine and a lower concentration of l-glutamine were previously identified as biomarkers of psoriasis.^53^ VIP score analysis based on the multivariate statistical analysis of the NMR metabolomics data shows a decrease in l-leucine and l-alanine and an increase in glutamine and glutamate at T1.

Moreover, significant variations in l-arginine, l-phenylalanine, l-aspartic acid, and l-proline concentrations at T1 were also observable.^53^ An interpretation of these metabolic changes according to the pathway and enrichment analysis (Figures 6 and 7) indicated that the regression of psoriasis was related to the correction of amino acid metabolic pathways, in particular, those of alanine, aspartate, and glutamate metabolism; arginine and proline metabolism; valine, leucine, and isoleucine degradation; valine, leucine, and isoleucine biosynthesis; and tyrosine metabolism. Therefore, these data, considered from a pathognomonic perspective, suggest that important modifications in amino acid and glycolysis pathways for psoriasis patients are ascribed to an increase in amino acid and energy demand for protein biosynthesis and keratinocyte hyperproliferation.^53^

NMR metabolomics data showed that choline levels were decreased in psoriasis patients at T1 compared to T0. The decrease in choline and nicotine concentrations is additional evidence that the regression of psoriasis corresponds to the regression of the inflammatory process: previous data showed high choline and nicotine levels in psoriasis patients due to mast cell degranulation.^52^

Additionally, we measured the concentration of the cytokines IL-4, TNF-α, INF-γ, IL-2, and IL-1β that are considered markers of inflammatory status. In agreement with previous scientific studies pointing to altered interleukin serum concentrations in psoriasis patients,^66,67^ we found decreased IL-2 and IL-1β concentrations at T1 compared to T0^52,53^ (Figure 3). The concentrations of IL-4, TNF-α, and INF-γ were not significantly decreased, probably due to the insufficient 4 week period to induce a significant variation of pro-inflammatory cytokine concentrations.

## Conclusions

Taken together, our data suggest that a low-calorie ketogenic diet can be considered a successful strategy and therapeutic option for the management of psoriasis. IL-2 and IL-1β, together with the concentrations of leucine, alanine, glutamine, glutamate, and choline, can be considered promising biomarkers for the early diagnosis and correct prognosis of psoriasis patients.

Our data suggest that a low-calorie ketogenic diet can be considered a successful strategy and therapeutic option for psoriasis management. IL-2 and IL-1β, together with the concentrations of l-leucine, l-alanine, l-glutamine, l-glutamate, and choline, can be considered promising biomarkers for the early diagnosis and correct prognosis of psoriasis patients. The dietary program is feasible, with high compliance, and safe. The main effects depend on reducing VAT, disrupting the inflammatory environment, and the source of inflammatory cytokines.

## Supporting Information Available

Figure S1: green line on top showing the accumulated variance explained and blue line underneath showing the variance explained by individual PC; Figure S2: pairwise score plots among the selected PCs; Figure S3: PLS-DA classification using different numbers of components; Table S1: important features identified by fold change and logarithmic fold change (log_2_(FC)) parameters calculated; Table S2: important features identified by *t*-test values, *P* values (threshold, <0.05), logarithmic *P* values and false discovery rate (FDR) parameters calculated for the most statistically significant compounds; Table S3: important features identified by PLS-DA and relative component VIP score; Table S4: results from quantitative enrichment analysis; Table S5: pathway analysis by Reactome and relative *P* value and number of hits; Table S6: clinical features of the patients during the follow-up (PDF)

## Abbreviations

PASI: psoriasis area and severity index
BSA: body surface area
DLQI: dermatology life quality index
BMI: body mass index
NMR: nuclear magnetic resonance
GH: growth hormone
HDL: high-density lipoprotein
LDL: low-density lipoprotein
AST: aspartate aminotransferase
γGT: gamma glutamyl transferase
HOMA-IR: homeostasis model assessment insulin resistance
VAT: visceral adipose tissue
AMFT: aortomesenteric fat thickness
CPMG: Carr–Purcell–Meiboom–Gill
HMDB: Human Metabolome Database
MVA: multivariate analysis
PCA: principal component analysis
PLS-DA: partial least-squares discriminant analysis
TSP: trimethylsilyl propionic-2,2,3,3-*d*_4_ acid, sodium salt
PRICONA: PRincipal COmponent Normalization Algorithm
ELISA: enzyme-linked immunosorbent assay
VIP: variable influence on projection
VAS: visual analogue scale
FDR: false discovery rate
PPAR: peroxisome proliferator-activated receptor

## References

[ref1] Takeshita J.; Gelfand J. M.; Li P.; Pinto L.; Yu X.; Rao P.; Viswanathan H. N.; Doshi J. A. Psoriasis in the US Medicare population: prevalence, treatment, and factors associated with biologic use. J. Invest. Dermatol. 2015, 135, 2955–2963. 10.1038/jid.2015.296. 26214380 PMC4549797

[ref2] Rachakonda T. D.; Schupp C. W.; Armstrong A. W. Psoriasis prevalence among adults in the United States. J. Am. Acad. Dermatol. 2014, 70, 512–516. 10.1016/j.jaad.2013.11.013. 24388724

[ref3] Gupta R.; Debbaneh M. G.; Liao W. Genetic epidemiology of psoriasis. Curr. Dermatol. Rep. 2014, 3, 61–78. 10.1007/s13671-013-0066-6. 25580373 PMC4285384

[ref4] Napolitano M.; Mastroeni S.; Fania L.; Pallotta S.; Fusari R.; Uras C.; Panebianco A.; Cavani A.; Sampogna F.; Abeni D. Sex- and gender-associated clinical and psychosocial characteristics of patients with psoriasis. Clin. Exp. Dermatol. 2020, 45, 705–711. 10.1111/ced.14218. 32170752

[ref5] Hagg D.; Sundstrom A.; Eriksson M.; Schmitt-Egenolf M. Severity of Psoriasis Differs Between Men and Women: A Study of the Clinical Outcome Measure Psoriasis Area and Severity Index (PASI) in 5438 Swedish Register Patients. Am. J. Clin. Dermatol. 2017, 18, 583–590. 10.1007/s40257-017-0274-0. 28342016 PMC5506504

[ref6] De Arruda L. H. F.; De Moraes A. P. F. The impact of psoriasis on quality of life. Br. J. Dermatol. 2001, 144, 33–36. 10.1046/j.1365-2133.2001.144s58033.x. 11501512

[ref7] Schadler E. D.; Ortel B.; Mehlis S. L. Biologics for the primary care physician: review and treatment of psoriasis. Disease-a-Month 2019, 65, 51–90. 10.1016/j.disamonth.2018.06.001. 30037762

[ref8] Brandon A.; Mufti A.; Sibbald R. G. Diagnosis and management of cutaneous psoriasis: a review. Adv. Skin Wound Care 2019, 32, 58–69. 10.1097/01.ASW.0000550592.08674.43. 30653184

[ref9] Hamminga E. A.; van der Lely A. J.; Neumann H. A. M.; Thio H. B. Chronic inflammation in psoriasis and obesity: implications for therapy. Med. Hypotheses 2006, 67, 768–773. 10.1016/j.mehy.2005.11.050. 16781085

[ref10] Armstrong A. W.; Harskamp C. T.; Armstrong E. J. diabetes, The association between psoriasis and obesity: a systematic review and meta-analysis of observational studies. Nutr. Diabetes 2012, 2, e54–e54. 10.1038/nutd.2012.26. 23208415 PMC3542430

[ref11] Carrascosa J.; Rocamora V.; Fernandez-Torres R.; Jimenez-Puya R.; Moreno J.; Coll-Puigserver N.; Fonseca E. Obesity and psoriasis: inflammatory nature of obesity, relationship between psoriasis and obesity, and therapeutic implications. Actas Dermo-Sifiliogr. 2014, 105, 31–44. 10.1016/j.ad.2012.08.003. 23177976

[ref12] Budu-Aggrey A.; Brumpton B.; Tyrrell J.; Watkins S.; Modalsli E. H.; Celis-Morales C.; Ferguson L. D.; Vie G. Å.; Palmer T.; Fritsche L. G. Evidence of a causal relationship between body mass index and psoriasis: A mendelian randomization study. PLoS Med. 2019, 16, 1–18. 10.1371/journal.pmed.1002739 PMC6354959 30703100

[ref13] Jensen P.; Zachariae C.; Christensen R.; Geiker N. R. W.; Schaadt B. K.; Stender S.; Hansen P. R.; Astrup A.; Skov L. Effect of weight loss on the severity of psoriasis: a randomized clinical study. JAMA Dermatol. 2013, 149, 795–801. 10.1001/jamadermatol.2013.722. 23752669

[ref14] Upala S.; Sanguankeo A. Effect of lifestyle weight loss intervention on disease severity in patients with psoriasis: a systematic review and meta-analysis. Int J. Obes. 2015, 39, 1197–1202. 10.1038/ijo.2015.64. 25920774

[ref15] Gisondi P.; Del Giglio M.; Di Francesco V.; Zamboni M.; Girolomoni G. Weight loss improves the response of obese patients with moderate-to-severe chronic plaque psoriasis to low-dose cyclosporine therapy: a randomized, controlled, investigator-blinded clinical trial. Am. J. Clin. Nutr. 2008, 88, 1242–1247. 10.3945/ajcn.2008.26427. 18996858

[ref16] Bardazzi F.; Balestri R.; Baldi E.; Antonucci A.; De Tommaso S.; Patrizi A. Correlation between BMI and PASI in patients affected by moderate to severe psoriasis undergoing biological therapy. Dermatol. Ther. 2010, 23, S14–S19. 10.1111/j.1529-8019.2009.01281.x. 20136916

[ref17] Castaldo G.; Galdo G.; Aufiero F. R.; Cereda E. Very low-calorie ketogenic diet may allow restoring response to systemic therapy in relapsing plaque psoriasis. Obes. Res. Clin. Pract. 2016, 10, 348–352. 10.1016/j.orcp.2015.10.008. 26559897

[ref18] Dupuis N.; Curatolo N.; Benoist J. F.; Auvin S. Ketogenic diet exhibits anti-inflammatory properties. Epilepsia 2015, 56, e95–e98. 10.1111/epi.13038. 26011473

[ref19] Franz M. J.; VanWormer J. J.; Crain A. L.; Boucher J. L.; Histon T.; Caplan W.; Bowman J. D.; Pronk N. P. Weight-loss outcomes: a systematic review and meta-analysis of weight-loss clinical trials with a minimum 1-year follow-up. J. Am. Diet. Assoc. 2007, 107, 1755–1767. 10.1016/j.jada.2007.07.017. 17904936

[ref20] Wycherley T. P.; Moran L. J.; Clifton P. M.; Noakes M.; Brinkworth G. D. Effects of energy-restricted high-protein, low-fat compared with standard-protein, low-fat diets: a meta-analysis of randomized controlled trials. Am. J. Clin. Nutr. 2012, 96, 1281–1298. 10.3945/ajcn.112.044321. 23097268

[ref21] Chaston T. B.; Dixon J. B. Factors associated with percent change in visceral versus subcutaneous abdominal fat during weight loss: findings from a systematic review. Int. J. Obes. 2008, 32, 619–628. 10.1038/sj.ijo.0803761. 18180786

[ref22] Castaldo G.; Rastrelli L.; Galdo G.; Molettieri P.; Aufiero F. R.; Cereda E. Aggressive weight loss program with a ketogenic induction phase for the treatment of chronic plaque psoriasis: a proof-of-concept, single-arm, open label clinical trial. Nutrition 2020, 74, 110757 10.1016/j.nut.2020.110757. 32222582

[ref23] Paoli A.; Rubini A.; Volek J. S.; Grimaldi K. A. Beyond weight loss: a review of the therapeutic uses of very-low-carbohydrate (ketogenic) diets. Eur. J. Clin. Nutr. 2014, 68, 641 10.1038/ejcn.2014.47. PMC3826507 23801097

[ref24] Ruskin D. N.; Svedova J.; Cote J. L.; Sandau U.; Rho J. M.; Kawamura M.; Boison D.; Masino S. A. Ketogenic diet improves core symptoms of autism in BTBR mice. PLoS One 2013, 8, 65021 10.1371/journal.pone.0065021. PMC3673987 23755170

[ref25] Hussain T. A.; Mathew T. C.; Dashti A. A.; Asfar S.; Al-Zaid N.; Dashti H. M. Effect of low-calorie versus low-carbohydrate ketogenic diet in type 2 diabetes. Nutrition 2012, 28, 1016–1021. 10.1016/j.nut.2012.01.016. 22673594

[ref26] Paoli A.; Mancin L.; Giacona M. C.; Bianco A.; Caprio M. Effects of a ketogenic diet in overweight women with polycystic ovary syndrome. J Transl Med 2020, 18, 104 10.1186/s12967-020-02277-0. 32103756 PMC7045520

[ref27] Paoli A.; Grimaldi K.; Toniolo L.; Canato M.; Bianco A.; Fratter A. Nutrition and acne: therapeutic potential of ketogenic diets. Skin Pharmacol. Physiol. 2012, 25, 111–117. 10.1159/000336404. 22327146

[ref28] Wells J.; Swaminathan A.; Paseka J.; Hanson C. Efficacy and Safety of a Ketogenic Diet in Children and Adolescents with Refractory Epilepsy-A Review. Nutrients 2020, 12, 1809 10.3390/nu12061809. PMC7353240 32560503

[ref29] Klement R. J.; Brehm N.; Sweeney R. A. Ketogenic diets in medical oncology: a systematic review with focus on clinical outcomes. Med Oncol 2020, 37, 14 10.1007/s12032-020-1337-2. 31927631

[ref30] Castellana M.; Conte E.; Cignarelli A.; Perrini S.; Giustina A.; Giovanella L.; Giorgino F.; Trimboli P. Efficacy and safety of very low calorie ketogenic diet (VLCKD) in patients with overweight and obesity: A systematic review and meta-analysis. Rev Endocr Metab Disord 2020, 21, 5–16. 10.1007/s11154-019-09514-y. 31705259

[ref31] Beckonert O.; Keun H. C.; Ebbels T. M. D.; Bundy J.; Holmes E.; Lindon J. C.; Nicholson J. K. Metabolic profiling, metabolomic and metabonomic procedures for NMR spectroscopy of urine, plasma, serum and tissue extracts. Nat. Protoc. 2007, 2, 2692–2703. 10.1038/nprot.2007.376. 18007604

[ref32] Monaco L.; Castaldo L.; Castaldo G.; Monaco M.; Di Tommaso L.; Stassano P. Aortomesenteric fat thickness with ultrasound predicts metabolic diseases in obese patients. Am. J. Med. Sci. 2014, 347, 8–13. 10.1097/MAJ.0b013e318288f795. 24366220

[ref33] Palisi A.; Grimaldi M.; Sabatini P.; Montoro P.; Scrima M.; Rodriquez M.; D’Ursi A. M. A serum nuclear magnetic resonance-based metabolomic signature of antiphospholipid syndrome. J. Pharm. Biomed. Anal. 2017, 133, 90–95. 10.1016/j.jpba.2016.11.002. 27829500

[ref34] Van Q. N.; Chmurny G. N.; Veenstra T. D. The depletion of protein signals in metabonomics analysis with the WET–CPMG pulse sequence. Biochem. Biophys. Res. Commun. 2003, 301, 952–959. 10.1016/S0006-291X(03)00079-2. 12589805

[ref35] Mo H.; Raftery D. Pre-SAT180, a simple and effective method for residual water suppression. J. Magn. Reson. 2008, 190, 1–6. 10.1016/j.jmr.2007.09.016. 17945521 PMC2662483

[ref36] Aranibar N.; Borys M.; Mackin N. A.; Ly V.; Abu-Absi N.; Abu-Absi S.; Niemitz M.; Schilling B.; Li Z. J.; Brock B.; Russell R. J. II; Tymiak A.; Reily M. D. NMR-based metabolomics of mammalian cell and tissue cultures. J. Biomol. NMR 2011, 49, 195–206. 10.1007/s10858-011-9490-8. 21373840

[ref37] Jupin M.; Michiels P. J.; Girard F. C.; Spraul M.; Wijmenga S. S. NMR metabolomics profiling of blood plasma mimics shows that medium- and long-chain fatty acids differently release metabolites from human serum albumin. J. Magn. Reson. 2014, 239, 34–43. 10.1016/j.jmr.2013.11.019. 24374750

[ref38] Weljie A. M.; Newton J.; Mercier P.; Carlson E.; Slupsky C. M. Targeted profiling: quantitative analysis of 1H NMR metabolomics data. Anal. Chem. 2006, 78, 4430–4442. 10.1021/ac060209g. 16808451

[ref39] Romano R.; Vilasi S.; Acernese F.; Canonico R.; Vilasi A.; Giordano G.; Barone F. Comparison of 1h-nmr spectra by normalisation algorithms for studying amyloid toxicity in cells. Int. J. Biomed. Eng. Technol. 2013, 13, 370–382. 10.1504/IJBET.2013.058540.

[ref40] Lemmerling P.; Vanhamme L.; Romano R.; Van Huffel S. A subspace time-domain algorithm for automated NMR spectral normalization. J. Magn. Reson. 2002, 157, 190–199. 10.1006/jmre.2002.2598. 12323137

[ref41] Rainaldi G.; Romano R.; Indovina P.; Ferrante A.; Motta A.; Indovina P. L.; Santini M. T. Metabolomics using 1H-NMR of apoptosis and Necrosis in HL60 leukemia cells: differences between the two types of cell death and independence from the stimulus of apoptosis used. Radiat. Res. 2008, 169, 170–180. 10.1667/RR0958.1. 18220461

[ref42] Santini M. T.; Romano R.; Rainaldi G.; Ferrante A.; Motta A.; Indovina P. L. Increases in 1H-NMR mobile lipids are not always associated with overt apoptosis: evidence from MG-63 human osteosarcoma three-dimensional spheroids exposed to a low dose (2 Gy) of ionizing radiation. Radiat. Res. 2006, 165, 131–141. 10.1667/RR3500.1. 16435912

[ref43] Grimaldi M.; Palisi A.; Rossi G.; Stillitano I.; Faiella F.; Montoro P.; Rodriquez M.; Palladino R.; D’Ursi A. M.; Romano R. Saliva of patients affected by salivary gland tumour: An NMR metabolomics analysis. J. Pharm. Biomed. Anal. 2018, 160, 436–442. 10.1016/j.jpba.2018.08.015. 30138814

[ref44] Wehrens H.; Franceschi P. Meta-statistics for variable selection: the R package BioMark. J. Stat. Software 2012, 51, 1–15.

[ref45] Measuring health-related quality of life in patients with mild to moderate eczema and psoriasis: clinical validity, reliability and sensitivity to change of the DLQI. Br. J. Dermatol. 1999, 141, 698–702. 10.1046/j.1365-2133.1999.03112.x. 10583119

[ref46] De Korte J.; Mombers F. M.; Bos J. D.; Sprangers M. A. In Quality of life in patients with psoriasis: a systematic literature review, Journal of Investigative Dermatology Symposium Proceedings, 2004; Elsevier: 2004; pp. 140–147. 10.1046/j.1087-0024.2003.09110.x 15083781

[ref47] Chong J.; Wishart D. S.; Xia J. Using metaboanalyst 4.0 for comprehensive and integrative metabolomics data analysis. Curr. Protoc. Bioinf. 2019, 68, e86 10.1002/cpbi.86. 31756036

[ref48] Wishart D. S.; Feunang Y. D.; Marcu A.; Guo A. C.; Liang K.; Vázquez-Fresno R.; Sajed T.; Johnson D.; Li C.; Karu N. HMDB 4.0: the human metabolome database for 2018. Nucleic Acids Res. 2018, 46, D608–D617. 10.1093/nar/gkx1089. 29140435 PMC5753273

[ref49] McKnight P. E.; Najab J. Mann-Whitney U Test*;* John Wiley & Sons 2010; p 1–1.

[ref50] Benjamini Y. Controlling the false discovery rate: a practical and powerful approach to multiple testing. J. Royal. Stat. Soc.: Ser. B 1995, 57, 289–300. 10.1111/j.2517-6161.1995.tb02031.x.

[ref51] Kanehisa M. In The KEGG database, Novartis Foundation Symposium, 2002; Wiley Online Library: 2002; pp. 91–100. 12539951

[ref52] Radosa J.; Dyck W.; Goerdt S.; Kurzen H. The cholinergic system in guttate psoriasis with special reference to mast cells. Exp. Dermatol. 2011, 20, 677–679. 10.1111/j.1600-0625.2011.01283.x. 21521372

[ref53] Kang H.; Li X.; Zhou Q.; Quan C.; Xue F.; Zheng J.; Yu Y. Br. J. Dermatol. 2017, 176, 713–722. 10.1111/bjd.15008. 27564527

[ref54] Croft D.; Mundo A. F.; Haw R.; Milacic M.; Weiser J.; Wu G.; Caudy M.; Garapati P.; Gillespie M.; Kamdar M. R.; Jassal B.; Jupe S.; Matthews L.; May B.; Palatnik S.; Rothfels K.; Birney E.; Hermjakob H.; Stein L.; D’Eustachio P. The Reactome pathway knowledgebase. Nucleic Acids Res. 2014, 42, D472–D477. 10.1093/nar/gkt1102. 24243840 PMC3965010

[ref55] Cuzick J. A Wilcoxon-type test for trend. Stat. Med. 1985, 4, 543–547. 10.1002/sim.4780040416. 4089356

[ref56] Caprio M.; Infante M.; Moriconi E.; Armani A.; Fabbri A.; Mantovani G.; Mariani S.; Lubrano C.; Poggiogalle E.; Migliaccio S.; Donini L. M.; Basciani S.; Cignarelli A.; Conte E.; Ceccarini G.; Bogazzi F.; Cimino L.; Condorelli R. A.; La Vignera S.; Calogero A. E.; Gambineri A.; Vignozzi L.; Prodam F.; Aimaretti G.; Linsalata G.; Buralli S.; Monzani F.; Aversa A.; Vettor R.; Santini F.; Vitti P.; Gnessi L.; Pagotto U.; Giorgino F.; Colao A.; Lenzi A. Very-low-calorie ketogenic diet (VLCKD) in the management of metabolic diseases: systematic review and consensus statement from the Italian Society of Endocrinology (SIE). J. Endocrinol. Invest. 2019, 42, 1365–1386. 10.1007/s40618-019-01061-2. 31111407

[ref57] Aronson P. J. Cases of psoriasis improved by lowering homocysteine using 4-7 mg folic acid, vitamins B6 and B12 previously worsened using 1-2 mg daily folic acid, B6 and B12 folic acid. J. Transl. Sci 2017, 3, 1–6.

[ref58] Qadim H.; Goforoushan F.; Nejad S.; Goldust M. Studying the calcium serum level in patients suffering from psoriasis. Pak. J. Biol. Sci 2013, 16, 291–294. 10.3923/pjbs.2013.291.294. 24498793

[ref59] Balta S.; Balta I.; Mikhailidis D. P.; Ozturk C.; Demirkol S.; Celik T.; Kilic S.; Demir M.; Iyisoy A. Bilirubin levels and their association with carotid intima media thickness and high-sensitivity C-reactive protein in patients with psoriasis vulgaris. Am. J. Clin. Dermatol. 2014, 15, 137–142. 10.1007/s40257-014-0069-5. 24696418

[ref60] Evers A. W. M.; Verhoeven E. W. M.; Kraaimaat F. W.; De Jong E. M. G. J.; De Brouwer S. J. M.; Schalkwijk J.; Sweep F. C. G. J.; Van De Kerkhof P. C. M. How stress gets under the skin: cortisol and stress reactivity in psoriasis. Br. J. Dermatol. 2010, 163, 986–991. 10.1111/j.1365-2133.2010.09984.x. 20716227

[ref61] Loftus T. M.; Lane M. D. Modulating the transcriptional control of adipogenesis. Curr. Opin. Genet. Dev. 1997, 7, 603–608. 10.1016/S0959-437X(97)80006-8. 9388775

[ref62] Lerner M. R.; Lerner A. B. Psoriasis and protein intake. Arch. Dermatol. 1964, 90, 217–225. 10.1001/archderm.1964.01600020085021. 14162332

[ref63] Rothberg S.; Crounse R. G.; Davis L. Jr.; Avogardo L.; Lamas J. The amino acid composition of protein fractions from normal and abnormal epidermis. J. Invest. Dermatol. 1965, 44, 320–325. 10.1038/jid.1965.56. 14290302

[ref64] Sitter B.; Johnsson M. K.; Halgunset J.; Bathen T. F. Metabolic changes in psoriatic skin under topical corticosteroid treatment. BMC dermatol. 2013, 13, 1–7. 10.1186/1471-5945-13-8. 23945194 PMC3751591

[ref65] Psychogios N.; Hau D. D.; Peng J.; Guo A. C.; Mandal R.; Bouatra S.; Sinelnikov I.; Krishnamurthy R.; Eisner R.; Gautam B.; Young N.; Xia J.; Knox C.; Dong E.; Huang P.; Hollander Z.; Pedersen T. L.; Smith S. R.; Bamforth F.; Greiner R.; McManus B.; Newman J. W.; Goodfriend T.; Wishart D. S. The human serum metabolome. PLoS One 2011, 6, e16957 10.1371/journal.pone.0016957. 21359215 PMC3040193

[ref66] Cai Y.; Xue F.; Quan C.; Qu M.; Liu N.; Zhang Y.; Fleming C.; Hu X.; Zhang H.-G.; Weichselbaum R.; Fu Y.-X.; Tieri D.; Rouchka E. C.; Zheng J.; Yan J. Signaling Pathway in Skin Inflammation and Psoriasis Pathogenesis. J. Invest. Dermatol. 2019, 139, 146–156. 10.1016/j.jid.2018.07.025. 30120937 PMC6392027

[ref67] Cataldi C.; Mari N. L.; Lozovoy M. A. B.; Martins L. M. M.; Reiche E. M. V.; Maes M.; Dichi I. Proinflammatory and anti-inflammatory cytokine profiles in psoriasis: use as laboratory biomarkers and disease predictors. Inflammation Res. 2019, 68, 557–567. 10.1007/s00011-019-01238-8. 31062065

