# Supplementary material for: Effect of Very-Low-Calorie Ketogenic Diet on Psoriasis Patients: A Nuclear Magnetic Resonance-Based Metabolomic Study
Source: J Proteome Res. 2020 Nov 9;20(3):1509–21. doi: 10.1021/acs.jproteome.0c00646 (PMC8016365; doi:10.1021/acs.jproteome.0c00646)
Supplement: Supplementary file 1 — pr0c00646_si_001.pdf [file pr0c00646_si_001.pdf]

Effect of very-low-calorie ketogenic diet on psoriasis patients: an NMR based metabolomic study

Giuseppe Castaldo[1], Imma Pagano[1,2], Manuela Grimaldi[2], Carmen Marino[2], Paola Molettieri[1], Angelo Santoro[2], Ilaria Stillitano[2], Rocco Romano[2], Paola Montoro[2], Anna Maria D'Ursi[2*], Luca Rastrelli[1,2*]

[1]NutriKeto_LAB Unisa - "San Giuseppe Moscati" National Hospital (AORN), Contrada Amoretta, 83100 Avellino (AV), Italy.

[2]Department of Pharmacy, University of Salerno, Via Giovanni Paolo II 132, 84084 Fisciano (SA), Italy.

*Corresponding Authors: Prof. Anna Maria D'Ursi, Ph.D. Associate Professor, DIFARMA, University of Salerno via Giovanni Paolo II, 132, 84084, Fisciano (SA) Tel +39089969748, e-mail: dursi@unisa.it; Luca Rastrelli, Department of Pharmacy, University of Salerno, Via Giovanni Paolo II 132, 84084 Fisciano (SA), Italy, Tel.: +39 089969766, e-mail address: dursi@unisa.it; rastrelli@unisa.it

Table of contents

- **Figure S1:** The green line on top shows the accumulated variance explained; the blue underneath line shows the variance explained by individual PC.

- **Figure S2:** Pairwise score plots among the selected PCs. The explained variance of each PC is shown in the corresponding diagonal cell. On the x-axis and y-axis are represented the position points in the Cartesian space of the individual samples.

- **Figure S3**: PLS-DA classification using different number of components. The red star indicates the best classifier.

- **Table S1:** Important features identified by Fold Change and logarithmic Fold Change (*log2(FC)*) parameters calculated.

- **Table S2:** Important features identified by *t-tests* values, *p-values* (Threshold <0.05), logarithmic *p-values* and False Discovery Rate (*FDR*) parameters calculated for the most statistically significative compounds.

- **Table S3**: Important features identified by PLS-DA and relative component VIP score.

- **Table S4:** Results from quantitative Enrichment Analysis.

- **Table S5:** Pathway analysis by Reactome and relative p-value and number of hits.

- **Table S6:** Clinical features of the patients during the follow-up.

Figure S1: The green line on top shows the accumulated variance explained; the blue underneath line shows the variance explained by individual PC.

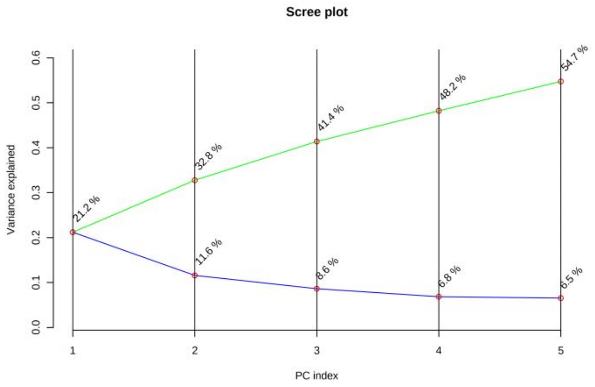

Figure S2: Pairwise score plots among the selected PCs. The explained variance of each PC is shown in the corresponding diagonal cell. On the x-axis and y-axis are represented the position points in the Cartesian space of the individual samples.

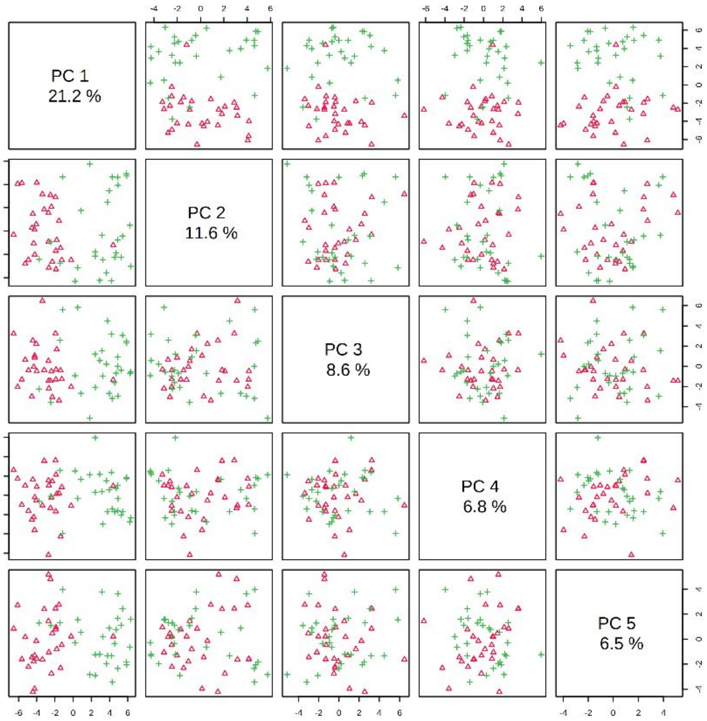

Figure S3: PLS-DA classification using different number of components. The red star indicates the best classifier.

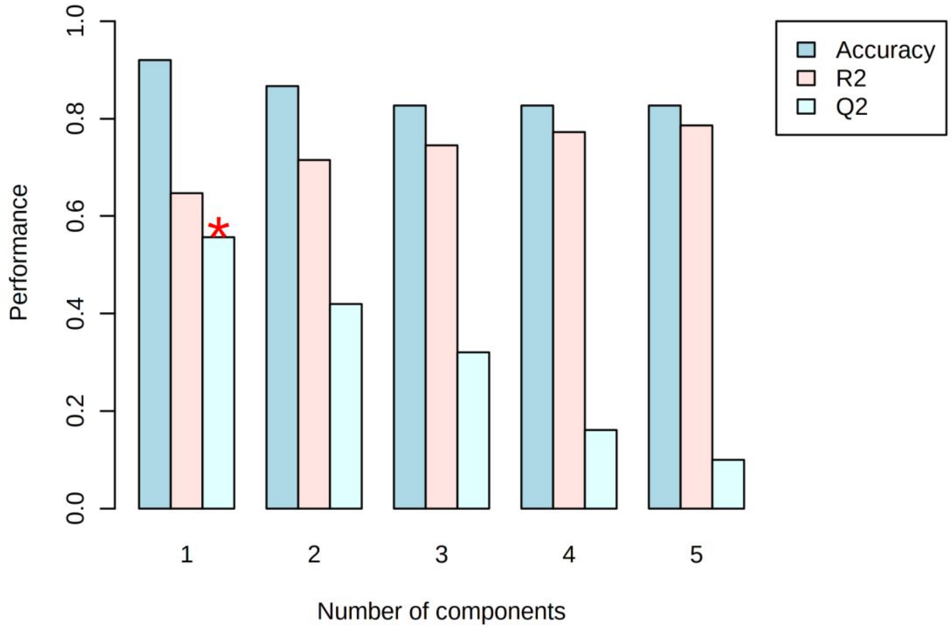

Table S1: Important features identified by Fold Change and logarithmic Fold Change (*log2(FC)*) parameters calculated.

**Compounds**	**Fold Change**	**log2(FC)**
L-Lysine	6.63	2.73
L-Histidine	5.55	2.47
Succinate	0.20	-2.29
Methionine	4.67	2.22
Formate	4.40	2.14
L-Serine	3.76	1.91
Malonate	3.69	1.88
Tryptophan	3.47	1.79
L-Ornithine	3.04	1.60
Tyrosine	2.60	1.37
L-Arginine	2.23	1.15
Isoleucine	0.45	-1.13
Choline	2.10	1.07
L-Glutamine	2.07	1.05

Table S2: Important features identified by *t-test* values, *P-values* (Threshold <0.05), logarithmic *P-values* and False Discovery Rate (*FDR*) parameters calculated for the most statistically significative compounds.

Compounds	t.stat	P-value	-log10(p)	FDR
Formate	12.6	4.4517e-18	17.351	1.7362e-16
Tryptophan	8.49	1.1764e-11	10.929	2.0211e-10
Tyrosine	8.42	1.5547e-11	10.808	2.0211e-10
L-Lysine	8.06	6.0830e-12	10.216	5.9309e-10
L-Histidine	7.72	2.1574e-10	9.6661	1.6828e-09
Methionine	7.61	3.3584e-10	9.4739	2.1829e-09
L-Arginine	6.40	3.3424e-08	7.4759	1.8622e-07
L-Lactic acid	-6.02	1.3854e-07	6.8584	6.7539e-07
L-Serine	5.78	3.4188e-07	6.4661	1.4815e-06
Choline	5.25	2.3648e-06	5.6262	9.2228e-06
Isoleucine	-5.00	5.7803e-06	5.2380	2.0494e-05
L-Ornithine	4.96	6.8468e-06	5.1645	2.2252e-05
Succinate	-4.30	6.7160e-06	4.1729	2.0148e-04
L-Glutamine	4.23	8.8084e-05	4.0551	2.4538e-04
Malonate	4.10	1.3621e-04	3.8658	3.5415e-04
L-Leucine	3.79	3.5944e-04	3.4444	8.7614e-04
Dimethyl sulfone	3.73	4.4553e-04	3.3511	1.0221e-02
Aspartate	-3.30	1.6732e-03	2.7765	3.6253e-02
L-Proline	3.24	1.9958e-03	2.6999	4.0966e-02
L-Glutamic acid	-2.79	7.1938e-02	2.1430	1.4028e-02
1-Methylhistidine	-2.45	1.7614e-02	1.7541	3.2712e-02

Table S3: Important features identified by PLS-DA and relative component VIP score.

Metabolite	Comp. 1	Comp. 2	Comp. 3	Comp. 4	Comp. 5
1-Methylhistidine	0.1313	0.1485	0.1522	0.2115	0.2153
2-Hydroxybutyric acid	0.3934	0.3770	0.4481	0.5699	0.5676
Acetic acid	0.1443	0.1593	0.1561	0.1553	0.1621
Betaine	0.2506	0.2429	0.2408	0.2370	0.2343
Acetoacetate	0.9722	1.0820	1.0609	1.0549	1.0476
Creatine	0.1839	0.2063	0.3000	0.2098	0.2491
Choline	1.7734	1.6961	1.6703	1.6466	1.6286
Glycine	0.3172	0.3294	0.4045	0.6112	0.6869
Formate	0.4538	0.4793	0.4711	0.4634	0.4580

L-Glutamic acid	0.8750	0.8365	0.8346	0.8262	0.8232
Tyrosine	0.3790	0.3941	0.4194	0.4210	0.4333
L-Phenylalanine	0.0882	0.0932	0.1335	0.1447	0.1592
L-Alanine	1.0361	1.0080	1.0042	0.9850	0.9844
L-Proline	0.5301	0.5073	0.5056	0.5059	0.5002
L-Threonine	0.2809	0.3276	0.3575	0.3529	0.3634
L-Asparagine	0.1972	0.2170	0.2582	0.2840	0.2933
Isoleucine	0.2348	0.2319	0.2310	0.2305	0.2988
L-Histidine	0.0657	0.5181	0.5868	0.6450	0.7339
L-Lysine	0.2026	0.2900	0.3330	0.3266	0.3252
L-Lactic acid	0.3376	0.4495	0.4843	0.4750	0.4816
Aspartate	0.0203	0.1164	0.1572	0.2593	0.2769
L-Ornithine	0.1171	0.1120	0.1342	0.1416	0.1480
Pyruvic acid	1.8077	1.7964	1.7649	1.7349	1.7181
Succinate	0.3608	0.3466	0.4474	0.4901	0.4845
3-Hydroxybutyric acid	4.3921	4.2296	4.1454	4.0732	4.0267
Creatinine	0.0136	0.1364	0.3756	0.3714	0.3951
L-Glutamine	0.3221	0.8027	0.7874	0.7826	0.8301
L-Leucine	2.4456	2.3559	2.3098	2.2717	2.2529
Methionine	0.3597	0.5952	0.5885	0.6053	0.5996
Isopropyl alcohol	0.5379	0.5264	0.5705	0.6015	0.6661
Valine	0.0247	0.0796	0.0820	0.2890	0.3140
Tryptophan	0.0403	0.5393	0.6037	0.6224	0.6263
Acetone	0.9952	0.9658	0.9476	0.9471	0.9400
Isobutyric acid	0.2358	0.2299	0.2622	0.2580	0.323
Methanol	0.0303	0.2234	0.5370	0.7658	0.8219
Propylene glycol	0.3722	0.6860	0.7365	0.7976	0.7912
Dimethyl sulfone	0.5128	0.6758	0.8479	0.8355	0.8293
D-Glucose	0.2285	0.2344	0.2420	0.2376	0.2667

Table S4: Results from quantitative Enrichment Analysis.

	Total Cmpd	Hits	Statistic Q	Expected Q	Raw p	Holm p	FDR
Fatty Acid Biosynthesis	35	3	47.89	1.75	2.40E-13	1.51E-11	1.51E-11
Glucose-Alanine Cycle	13	4	20.32	1.75	3.40E-07	2.11E-05	1.06E-05
Phospholipid Biosynthesis	29	1	35.40	1.75	8.41E-07	5.13E-05	1.06E-05
Phosphatidylcholine Biosynthesis	14	1	35.40	1.75	8.41E-07	5.13E-05	1.06E-05
Phosphatidylethanolamine Biosynthesis	12	1	35.40	1.75	8.41E-07	5.13E-05	1.06E-05
Valine, Leucine and Isoleucine	60	6	15.95	1.75	2.55E-06	1.48E-04	2.67E-05

Degradation							
Cysteine Metabolism	26	2	22.22	1.75	3.08E-06	1.76E-04	2.78E-05
Urea Cycle	29	6	12.06	1.75	5.19E-06	2.91E-04	4.09E-05
Betaine Metabolism	21	3	17.12	1.75	1.00E-05	5.53E-04	7.03E-05
Pyruvate Metabolism	48	4	16.65	1.75	1.18E-05	6.36E-04	7.42E-05
Gluconeogenesis	35	3	21.90	1.75	1.40E-05	7.42E-04	7.50E-05
Pyruvaldehyde Degradation	10	1	28.38	1.75	1.67E-05	8.69E-04	7.50E-05
Glycolysis	25	2	25.50	1.75	1.73E-05	8.81E-04	7.50E-05
Transfer of Acetyl Groups into Mitochondria	22	2	25.50	1.75	1.73E-05	8.81E-04	7.50E-05
Citric Acid Cycle	32	2	18.55	1.75	1.78E-05	8.81E-04	7.50E-05
Warburg Effect	58	6	11.46	1.75	2.43E-05	1.17E-03	9.56E-05
Amino Sugar Metabolism	33	4	13.25	1.75	2.88E-05	1.36E-03	1.07E-04
Alanine Metabolism	17	4	12.97	1.75	3.60E-05	1.66E-03	1.26E-04
Glycine and Serine Metabolism	59	9	8.73	1.75	4.70E-05	2.11E-03	1.56E-04
Tryptophan Metabolism	60	4	12.82	1.75	6.25E-05	2.75E-03	1.97E-04
Glutamate Metabolism	49	7	9.25	1.75	7.70E-05	3.31E-03	2.31E-04
Phenylalanine and Tyrosine Metabolism	28	4	11.05	1.75	4.54E-04	1.91E-02	1.30E-03
Tyrosine Metabolism	72	4	9.98	1.75	5.77E-04	2.36E-02	1.58E-03
Selenoamino Acid Metabolism	28	1	18.72	1.75	6.94E-04	2.78E-02	1.82E-03
Methionine Metabolism	43	4	9.61	1.75	1.15E-03	4.48E-02	2.90E-03
Ketone Body Metabolism	13	3	9.12	1.75	1.59E-03	6.06E-02	3.86E-03
Ammonia Recycling	32	7	6.11	1.75	2.95E-03	1.09E-01	6.88E-03
Folate Metabolism	29	2	10.88	1.75	4.18E-03	1.51E-01	9.41E-03
Arachidonic Acid Metabolism	69	1	11.53	1.75	9.12E-03	3.19E-01	1.98E-02
Malate-Aspartate Shuttle	10	2	8.57	1.75	1.17E-02	3.96E-01	2.45E-02
Lysine Degradation	30	2	7.78	1.75	1.23E-02	4.06E-01	2.50E-02
Butyrate Metabolism	19	2	7.24	1.75	1.38E-02	4.42E-01	2.72E-02
Glutathione Metabolism	21	3	6.77	1.75	1.60E-02	4.97E-01	2.99E-02
Propanoate Metabolism	42	3	6.19	1.75	1.61E-02	4.97E-01	2.99E-02
Pterine Biosynthesis	29	1	9.01	1.75	2.20E-02	6.39E-01	3.51E-02
Steroid Biosynthesis	48	1	9.01	1.75	2.20E-02	6.39E-01	3.51E-02
Androgen and Estrogen Metabolism	33	1	9.01	1.75	2.20E-02	6.39E-01	3.51E-02
Androstenedione Metabolism	24	1	9.01	1.75	2.20E-02	6.39E-01	3.51E-02
Catecholamine Biosynthesis	20	1	8.98	1.75	2.23E-02	6.39E-01	3.51E-02
Thyroid hormone synthesis	13	1	8.98	1.75	2.23E-02	6.39E-01	3.51E-02
Nicotinate and Nicotinamide Metabolism	37	2	5.37	1.75	4.89E-02	1.00E+00	7.51E-02
Aspartate Metabolism	35	5	3.34	1.75	1.01E-01	1.00E+00	1.52E-01
Arginine and Proline Metabolism	53	7	2.98	1.75	1.30E-01	1.00E+00	1.90E-01
Threonine and 2-Oxobutanoate Degradation	20	1	3.56	1.75	1.56E-01	1.00E+00	2.12E-01
Sphingolipid Metabolism	40	1	3.47	1.75	1.62E-01	1.00E+00	2.12E-01
Galactose Metabolism	38	1	3.47	1.75	1.62E-01	1.00E+00	2.12E-01

Lactose Synthesis	20	1	3.47	1.75	1.62E-01	1.00E+00	2.12E-01
Lactose Degradation	9	1	3.47	1.75	1.62E-01	1.00E+00	2.12E-01
Histidine Metabolism	43	3	3.02	1.75	1.71E-01	1.00E+00	2.20E-01
Purine Metabolism	74	4	2.79	1.75	1.83E-01	1.00E+00	2.29E-01
Beta-Alanine Metabolism	34	3	2.85	1.75	1.85E-01	1.00E+00	2.29E-01
Oxidation of Branched Chain Fatty Acids	26	1	1.91	1.75	3.00E-01	1.00E+00	3.50E-01
Mitochondrial Electron Transport Chain	19	1	1.91	1.75	3.00E-01	1.00E+00	3.50E-01
Phytanic Acid Peroxisomal Oxidation	26	1	1.91	1.75	3.00E-01	1.00E+00	3.50E-01
Biotin Metabolism	8	1	1.10	1.75	4.33E-01	1.00E+00	4.82E-01
Pyrimidine Metabolism	59	1	1.09	1.75	4.36E-01	1.00E+00	4.82E-01
Phenylacetate Metabolism	9	1	1.09	1.75	4.36E-01	1.00E+00	4.82E-01
Spermidine and Spermine Biosynthesis	18	2	1.08	1.75	5.45E-01	1.00E+00	5.85E-01
Porphyrin Metabolism	40	1	0.62	1.75	5.57E-01	1.00E+00	5.85E-01
Bile Acid Biosynthesis	65	1	0.62	1.75	5.57E-01	1.00E+00	5.85E-01
Ethanol Degradation	19	1	0.57	1.75	5.75E-01	1.00E+00	5.94E-01
Carnitine Synthesis	22	3	1.01	1.75	5.93E-01	1.00E+00	6.03E-01
Methylhistidine Metabolism	4	1	0.02	1.75	9.08E-01	1.00E+00	9.08E-01

Table S5: Pathway analysis by Reactome and relative p-value and number of hits.

Pathway name		Entities		
	found	ratio	P-value	FDR*
Amino acid transport across the plasma membrane	17 / 34	0.017	1.11e-16	4.44e-15
tRNA Aminoacylation	16 / 28	0.014	1.11e-16	4.44e-15
Mitochondrial tRNA aminoacylation	16 / 28	0.014	1.11e-16	4.44e-15
Cytosolic tRNA aminoacylation	16 / 28	0.014	1.11e-16	4.44e-15
Transport of inorganic cations/anions and amino acids/oligopeptides	21 / 63	0.032	1.11e-16	4.44e-15
Na+/Cl- dependent neurotransmitter transporters	15 / 31	0.016	1.11e-16	4.44e-15
Transport of bile salts and organic acids, metal ions and amine compounds	21 / 86	0.043	1.11e-16	4.44e-15
SLC-mediated transmembrane transport	25 / 180	0.091	4.44e-16	1.55e-14
SLC transporter disorders	18 / 90	0.045	7.89e-14	2.45e-12
Translation	16 / 65	0.033	1.26e-13	3.53e-12
Disorders of transmembrane transporters	18 / 108	0.054	1.69e-12	4.24e-11
Defective SLC6A19 causes Hartnup disorder (HND)	9 / 13	0.007	8.03e-12	1.69e-10
Defective SLC6A19 causes Hartnup disorder (HND)	9 / 13	0.007	8.03e-12	1.69e-10
Transport of small molecules	25 / 280	0.141	1.13e-11	2.26e-10
Metabolism of amino acids and derivatives	26 / 318	0.16	2.32e-11	4.18e-10
Variant SLC6A14 may confer susceptibility towards obesity	9 / 15	0.008	2.82e-11	4.80e-10
Tryptophan catabolism	11 / 35	0.018	1.30e-10	2.09e-09
Phenylalanine and tyrosine metabolism	8 / 33	0.017	4.11e-07	6.17e-06
Ketone body metabolism	6 / 19	0.01	2.98e-06	4.17e-05
Metabolism of proteins	18 / 277	0.139	4.73e-06	6.62e-05

Table S6: Clinical features of the patients during the follow-up.

Parameter	Baseline T0		4 Weeks T1		*P value*[b]
	Mean	SD[a]	Mean	SD[a]	
Weight, kg	85.13	19.81	76.51	17.58	0.062
Height, cm	165.81	9.65			
BMI, kg/m^2	30.82	5.96	27.79	5.23	0.839
**Diastolic blood pressure, mm Hg**	**88.27**	**24.62**	**77.40**	**24.23**	**0.009**
**Systolic blood pressure, mm Hg**	**109.40**	**25.22**	**98.50**	**20.22**	**0.026**
Waist, cm	100.13	17.60	90.73	20.40	0.072
**Hip, cm**	**111.03**	**12.56**	**104.15**	**11.62**	**0.032**
Hemoglobin, g/dl	14.51	1.14	14.30	0.84	0.407
**Lymphocytes, mmc**	**2.02**	**0.73**	**1.63**	**0.53**	**0.029**
Hematocrit, %	42.77	3.19	42.38	2.51	0.668
Glycated hemoglobin, %	5.59	0.68	5.32	0.54	0.054
**Glycemia, mg/dL**	**90.60**	**17.36**	**77.23**	**12.80**	**0.001**
Azotemia, mg/dL	32.50	7.75	29.77	10.33	0.115
Creatinine, mg/dL	0.76	0.21	0.73	0.21	0.321
Uricemia, mg/dL	5.02	1.71	4.30	1.41	0.080
**Total cholesterol, mg/dL**	**193.40**	**31.34**	**144.20**	**27.13**	**0.0001**
**Low-density lipoprotein (LDL), mg/dL**	**122.53**	**24.94**	**83.37**	**25.48**	**0.0001**
High-density lipoprotein (HDL), mg/dL	52.07	18.23	44.20	14.22	0.079
Triglycerides, mg/dL	130.30	81.89	96.93	40.38	0.124
**Apolipoprotein A1 (Apo A1), mg/dL**	**140.73**	**31.28**	**114.10**	**24.46**	**0.001**
**Apolipoprotein B (Apo B), mg/dL**	**98.57**	**19.18**	**81.83**	**16.66**	**0.002**
**Prothrombin time (PT), %**	**1.08**	**0.60**	**1.05**	**0.08**	**0.002**
**Partial thromboplastin time (PTT), s**	**1.05**	**0.36**	**1.06**	**0.12**	**0.010**
**International normalized ratio (INR)**	**1.09**	**0.54**	**1.06**	**0.13**	**0.006**
Alkaline phosphatase (ALP), U/l	68.87	24.65	59.70	19.93	0.058
**Aspartate transaminase (AST), U/l**	**20.93**	**7.21**	**24.30**	**6.86**	**0.035**
Alanine transaminase (ALT), U/l	25.33	15.48	25.97	14.21	0.610
**Gamma glutamyl transferase (γGT), U/l**	**28.57**	**35.83**	**14.03**	**9.93**	**0.002**
Creatine kinase (CK), U/l	120.53	62.37	140.90	96.98	0.429
Lactate dehydrogenase (LDH), U/l	400.60	127.60	366.83	94.79	0.308
**Direct bilirubin, mg/dl**	**0.18**	**0.08**	**0.23**	**0.10**	**0.019**
Total bilirubin, mg/dl	0.53	0.31	0.53	0.33	0.881
**Choline esterase, (CHE), UI/l**	**8535.63**	**2096.80**	**7560.50**	**2265.25**	**0.036**
**Sideremia, µg/dL**	**93.13**	**36.85**	**72.97**	**15.15**	**0.017**
Total protein, g/L	6.99	0.45	6.97	0.39	0.795
Albumin, g/dL	4.40	0.27	4.46	0.27	0.348

Sodium, mEq/L	139.97	2.65	139.03	2.85	0.228
Potassium, mEq/L	4.55	0.38	4.63	0.37	0.462
**Calcium, mg/dL**	**9.41**	**0.40**	**9.74**	**0.53**	**0.005**
Magnesium, mEq/L	2.11	0.15	2.05	0.17	0.121
**Phosphorus, mg/dL**	**3.23**	**0.55**	**4.58**	**5.58**	**0.003**
**Fibrinogen, mg/dL**	**315.77**	**47.82**	**333.27**	**63.16**	**0.223**
Eritro-sedimentation velocity (VES), mm/h	16.57	9.28	16.13	10.56	0.796
C-reactive protein (CRP), mg/L	3.07	1.20	2.55	1.35	0.143
Free triiodothyronine (FT3), pg/ml	3.88	0.45	3.65	0.60	0.117
Free thyroxine (FT4), ng/dl	1.12	0.17	1.16	0.17	0.211
Thyrotropin (TSH), mlU/L	1.58	0.96	10.06	5.70	0.501
**Folic acid, ng/mL**	**5.52**	**2.26**	**10.66**	**6.22**	**0.0001**
**Vitamin B12, ng/L**	**294.17**	**155.15**	**460.17**	**218.56**	**0.001**
**Insulin, mcUI/ml**	**11.53**	**8.45**	**6.07**	**4.19**	**0.006**
**Omeostasis model assessment of insulin resistance, HOMA-IR**	**2.72**	**1.73**	**1.22**	**1.00**	**0.003**
**Cortisol, µg/dl**	**9.64**	**3.44**	**11.57**	**2.88**	**0.014**
Growth Hormone (GH), ng/ml	*0.77*	*1.70*	*2.25*	*2.60*	*0.0001*
Homocysteine, µmol/L	*17.09*	*8.59*	*14.58*	*3.93*	*0.003*
Aortomesenteric fat thickness (AMFT), mm	16.99	8.34	13.61	7.35	0.050
**PASI**	**8.69**	**7.13**	**4.10**	**3.65**	**0.007**
**DLQI**	**12.83**	**8.69**	**3.70**	**4.31**	**0.0001**
BSA, %	16.02	11.62	8.68	4.01	0.073
**VAS pruritus**	**43.67**	**36.36**	**14.41**	**11.01**	**0.003**
**VAS pain**	**37.83**	**33.85**	**11.07**	**8.18**	**0.003**
